# Functionalization of polyethylene with hydrolytically-stable ester groups†

**DOI:** 10.1039/d3ra05024f

**Published:** 2023-08-10

**Authors:** Susi Hervàs-Arnandis, Brenda Palomar-de Lucas, Cristina Bilanin, Paloma Mingueza-Verdejo, Mónica Viciano, Judit Oliver-Meseguer, Antonio Leyva-Pérez

**Affiliations:** a Instituto de Tecnología Química (UPV–CSIC), Universidad Politècnica de València–Consejo Superior de Investigaciones Científicas Avda. de los Naranjos s/n 46022 Valencia Spain joliverm@itq.upv.es anleyva@itq.upv.es; b AIMPLAS, València Parc Tecnològic C/Gustave Eiffel, 4 46980 Paterna Valencia Spain

## Abstract

Low-density (LD) and high-density polyethylene (HDPE), recycled or not, incorporates up to 7 wt% of ester groups after reacting either with ethyl diazoacetate (EDA) under catalytic and solvent free-reaction conditions, or with maleic anhydride (MA) and acrylates (AC) under catalytic radical conditions. The resulting upcycled polyethylene esters are hydrolytically stable at extreme pH (0–14) and can be further transformed into carboxylic acids, carboxylates, other esters and amides.

## Abstract 2

Polyethylene is functionalized with covalently anchored ester groups at high loadings, showing resistance to hydrolysis in water at extreme pHs and enabling further functionalizations.

## Graphical abstract

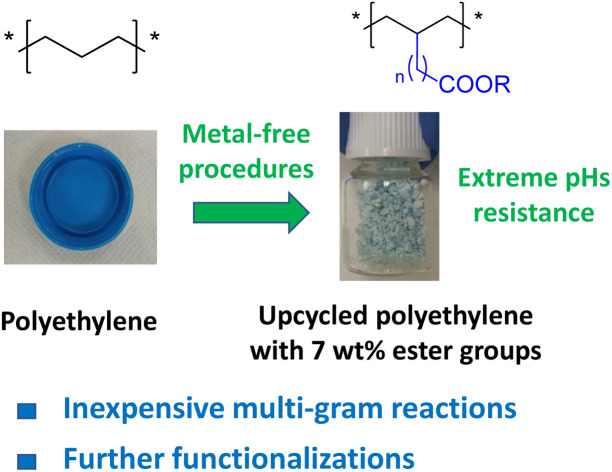

## 1 Introduction

Polyethylene (PE) is the longest linear alkane known, with an estimated annual production of >100 million tonnes, which accounts for 34% of the total plastics market. This extremely cheap and very long alkane is used on a daily basis in bags, caps, *etc.* However, its disposal has created a significant environmental problem, due in large part to the lack of upcycling strategies.^1^ Developed countries recycle less than 20% of the PE that is manufactured and consumed, and the rest is incinerated or ends up in the sea (Fig. S1†).^2^ Therefore, there is a need not only for an economic but also for an environmental solution, in short of sustainability, in order to look for other uses of PE.

**Fig. 1:**
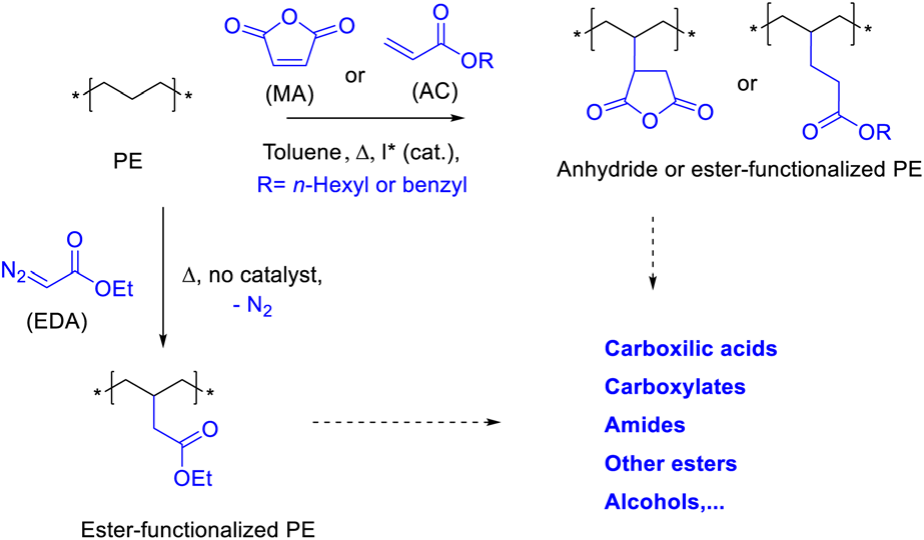
Proposed functionalization of polyethylene (PE) with ethyl diazoacetate (EDA), maleic anhydride (MA) or acrylates (AC), and potential further transformations.

Chemical upcycling is an attractive strategy to reuse PE.^3^ However, activation of the long methylene (CH_2_) PE chains under mild, scalable and economically viable conditions is still a challenge. Fig. 1 shows the three approaches here proposed to upcycle PE, which consist in reacting PE with either carbene esters (EDA), maleic anhydride (MA) or acrylates (AC), to generate a very versatile material in terms of further functionalization. The new PE esters are extremely long, 10–100 times longer than any alkyl chain esters currently on the market.^4^ Two of the synthetic approaches here studied are not new and have been reported in the past under reactions conditions which serve as starting point to study the functionalization reactions.^5^ However, it must be said that the previously reported conditions are far from being efficient or scalable to the required amounts of PE to be treated. On one hand, the reaction of EDA with PE has been carried out with organometallic catalysts,^5*c*^ since the uncatalyzed reaction yields <1% of functionalized polymer. The use of organometallic catalysts for EDA incorporation implies further purification steps, such as separation of the metal, and a perhaps unaffordable increase in price. On the other hand, the incorporation of MA has been achieved sometimes with loadings <0.5%,^5*d*^ and more importantly, the radical initiators employed generate extensive cross-linking PE products.^6^ PE is also extremely resistant to oxidizing and reducing agents,^7^ thus we discarded these approaches. Here, we show scalable and relatively cheap reaction protocols which enable the incorporation to PE of up to 7 wt% of ester groups. The direct coupling of acrylates to PE has, to our knowledge, not being reported yet.

Hydrolytically stable esters are high (>10) and low (<5) pH-resistant esters, essential in industrial applications where the material needs to be in continuous contact with water at extreme pHs, such as lubricants for acid water pressure pumps or hair softeners (pH > 13), to name a few.^4*b*^ Lubricants based on esters of long linear chains are a reality in the worldwide market. Since the stability of an ester against hydrolysis is mainly determined by the impediment exerted on the water molecules by the surrounding structure, long aliphatic chains are good candidates to repel protons or hydroxyls present in aqueous solutions.^8^ This is the reason why industrial manufacturing of hydrolytically stable esters is based on the esterification reaction of acids and alcohols with aliphatic chains as long as 20 to 40 carbon atoms, which requires much more severe production conditions than regular esters and, besides, uses starting materials from the fine chemical and not from the petrochemical or biomass industry,^9^ with the corresponding further increase in price. These drawbacks make to the chemical industry opt for poly(α-olefin)s as hydrolytically stable lubricating agents, much more polluting and less efficient than esters (the formed biodegrades much slower). All the above comments strongly suggest that a new ester material based on the extremely cheap and alkyl long chains, *i.e.* PE, will be of interest in the market and open new opportunities in the field. It is worthy to comment here that the resulting PE ester should not be confused with the currently marketed PE esters produced by cross-polymerization of ethylene and acrylates, since the latter has alternating ethane and ester groups in the linear chain, so that they do not have long aliphatic chains but quite the opposite, polar compounds, and they show low resistance to hydrolysis (*e.g.* polyethylene terephtalate, PET).

## 2 Results and discussion

### 2.1 Functionalization of PE with ethyl diazoacetate (EDA)

**Fig. 2:**
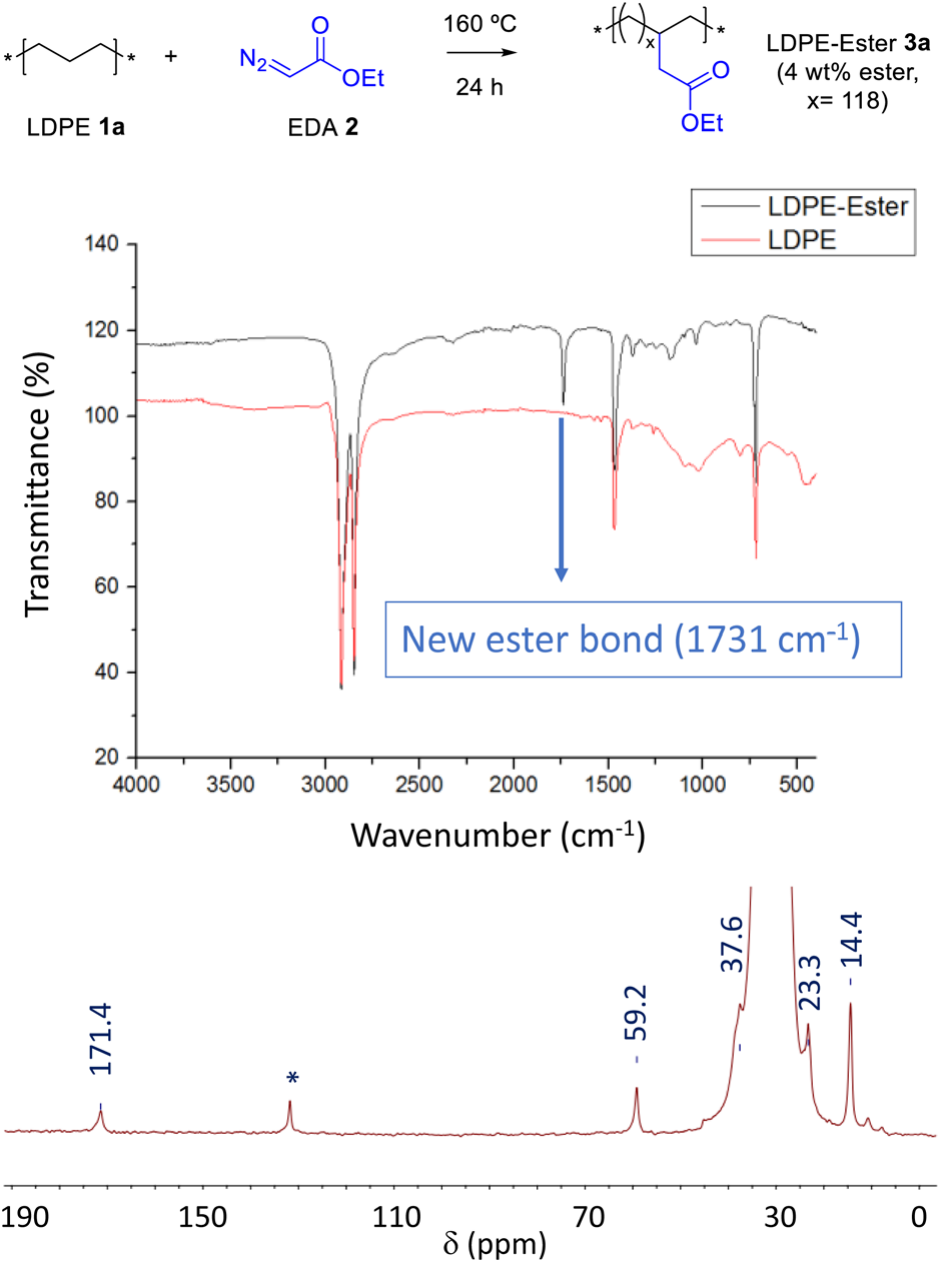
(Top) Synthesis of the ester-functionalized LDPE-3a with EDA. (Middle) FTIR spectra, before and after reaction. (Bottom) SS-MAS ^13^C NMR spectrum of LDPE-ester 3a. The signal at 131.0 ppm (*) is tentatively assigned to *in situ* formed PE alkenes.

Carbenes are carbon atoms with a defect of two instead of only one charge, thus, unlike radicals, they can act as nucleophiles and electrophiles at the same time.^10^ This feature can be used to remove hydrogen atoms from the PE CH_2_ backbone and add the ester functional group without modifying the native PE chain.^11^Fig. 2 shows that pristine low-density PE (LDPE, 1a) readily reacts with ethyl diazoacetate (EDA, 2) at 160 °C, without any catalyst, solvent or additive, to give the new PE material LDPE-ester 3a having pending ester groups in the structure, as indicated by the appearance of a signature peak at 1731 cm^−1^ in the corresponding Fourier transform-infrared (FT-IR) spectrum (see Table S1† for complete FT-IR signal assignments). An extensive washing of the material with the appropriate solvent is key to completely eliminate the EDA self-coupling by-products, which otherwise will give false anchoring ester signals, an issue not fully investigated in previous reports as far as we know. According to our results (Table S2†), aprotic solvents are the more suitable solvents for the washing operation, under agitation conditions at room temperature during 18 hours. Other solvents do not extract completely the EDA by-products, such as acetonitrile, water, *N*,*N*-dimethylformamide (DMF) and dimethyl sulfoxide (DMSO), and others such as tetrahydrofuran (THF) and 2-methyltetrahydrofuran dissolves the polymer at 70 °C. Gas chromatography coupled to mass spectrometry (GC-MS) confirmed not only the consumption of EDA 2 during reaction but also the washings of the EDA by-products from the isolated LDPE-ester 3a. The characteristic signal of ethyl ester in FT-IR (Fig. 2 above) is accompanied by the rest of signals of pristine PE, which indicates that the original CH_2_ backbone of the material remains unaltered. A calibration of the IR instrument with lauryl ester (Fig. S2†) and also quantitative ^1^H nuclear magnetic resonance experiments (NMR, Fig. S3†) show that the new PE material has incorporated around 4 wt% of ethyl ester groups.^12^ Scanning electron microscopy (SEM) images show that the intricated and stringy structure of LDPE 1a remains in the treated material 3a (Fig. S4†). ^1^H and ^13^C nuclear magnetic resonance (NMR) measurements of LDPE-ester 3a in liquid phase, dissolved in deuterated toluene and recorded in the NMR tube at 70–95 °C, confirm the covalent anchoring of the methylene ester groups (Fig. S3†). However, due to the low amount of LDPE-ester 3a able to be dissolved in toluene, the ester carbonyl peak did not appear in the ^13^C NMR spectrum. Thus, in order to fully confirm the anchoring of the ester group to the PE material, the solid-state magic angle spinning (SS-MAS) ^13^C NMR of LDPE-ester 3a was recorded. The spectrum in Fig. 2 shows the signal at 171.4 ppm corresponding to the ester carbonyl group, together with the characteristic signals of the neighboring CH_2_ (60.0 ppm), the CH_2_ backbone (30.5 ppm) and the ending CH_3_ groups (14.7 ppm). The signal at 36.1 ppm corresponds to the typical CH substitutions present in LDPE, and the signal at 131.0 ppm is tentatively assigned here to *in situ* formed PE alkenes. With this characterization in hand, we can calculate that the ester loading in LDPE-ester 3a corresponds to a free-to-functionalized CH_2_ relationship of 118, in other words, LDPE-ester 3a has one pending ester group each 118 methylene units, in average. A thermogravimetric analysis (TGA) shows that the LDPE-ester 3a material is stable up to 300 °C and then follows the typical decomposition pattern of LDPE (Fig. S5†).

**Fig. 3:**
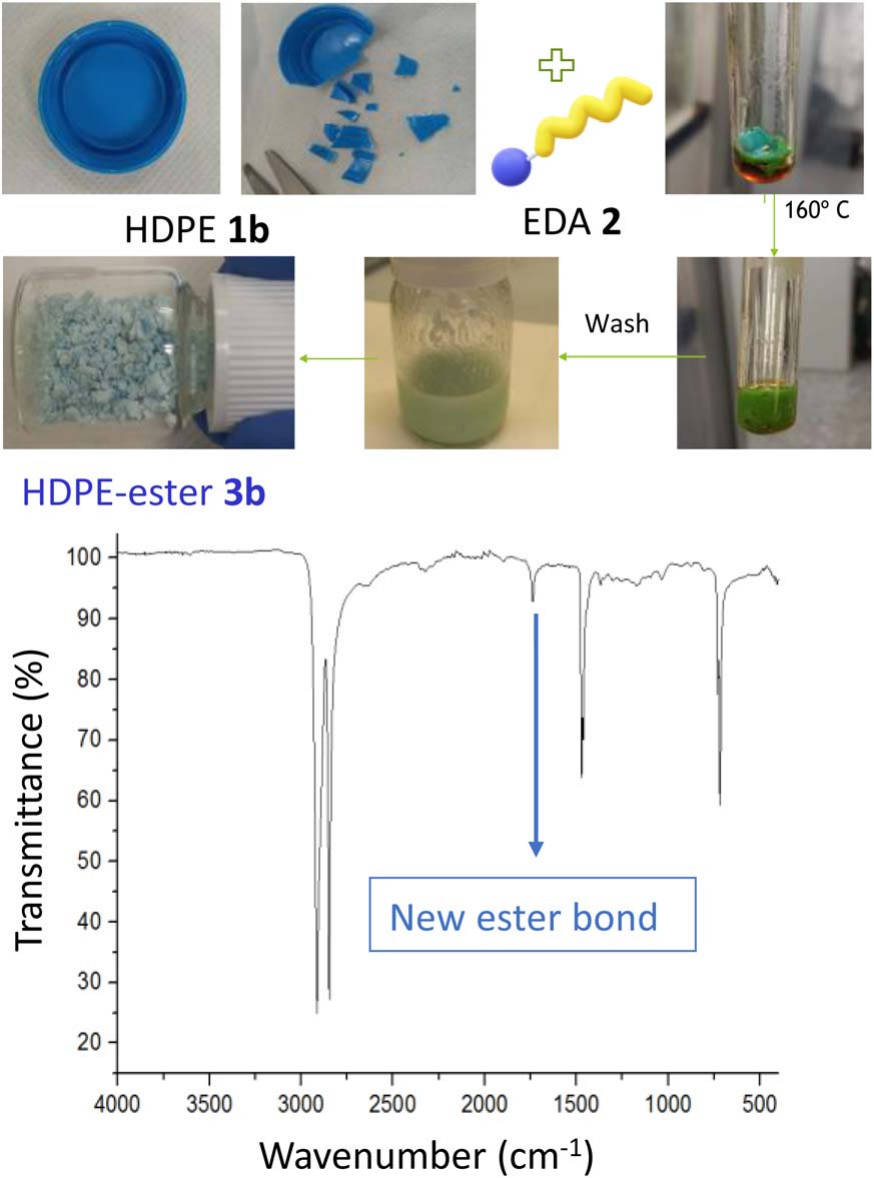
Esterification of a recycled HDPE 1b blue cap, with the corresponding photos of the process (top), and the FT-IR spectrum of the final HDPE-ester 3b material (bottom).

Fig. 3 shows the esterification reaction of recycled PE, in this case of a blue bottle cap made of high-density polyethylene (HDPE 1b). The functionalization was carried out after cutting in small pieces the HDPE cap, with a scissors, an putting the pieces under the same conditions described above for pristine LDPE 1a. After extensive washings, the resulting HDPE-ester 3b material preserves the blue color of the recycled cap but with ester groups covalently anchored on the polymeric structure, as assessed by the new band at 1715 cm^−1^ in the FT-IR spectrum. The original HDPE structure remains untouched, according to FT-IR (Fig. S6†). These results strongly indicate that both LDPE and HDPE are reactive towards the esterification reaction, and that post-consumer HDPE admits the covalent attachment of ester groups, thus most recycled PE materials could be successful for EDA functionalization. It is worthy to comment here that EDA is the most industrially used carbene, manufactured in multi-kilogram amounts,^13^ although of course with the inherent risks associated to such a reactive molecule.^13*b*^

### 2.2 Hydrolysis of LDPE-ester 3a

**Fig. 4:**
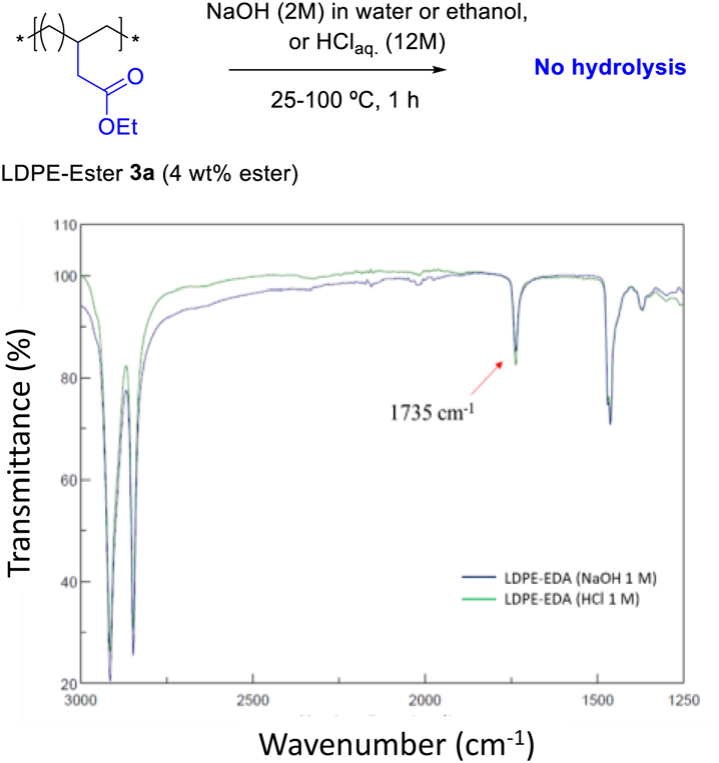
FT-IR spectra of LDPE-ester 3a treated with concentrated aqueous NaOH or HCl during 1 h, showing resistance towards hydrolysis at different pHs (0–14).

**Fig. 5:**
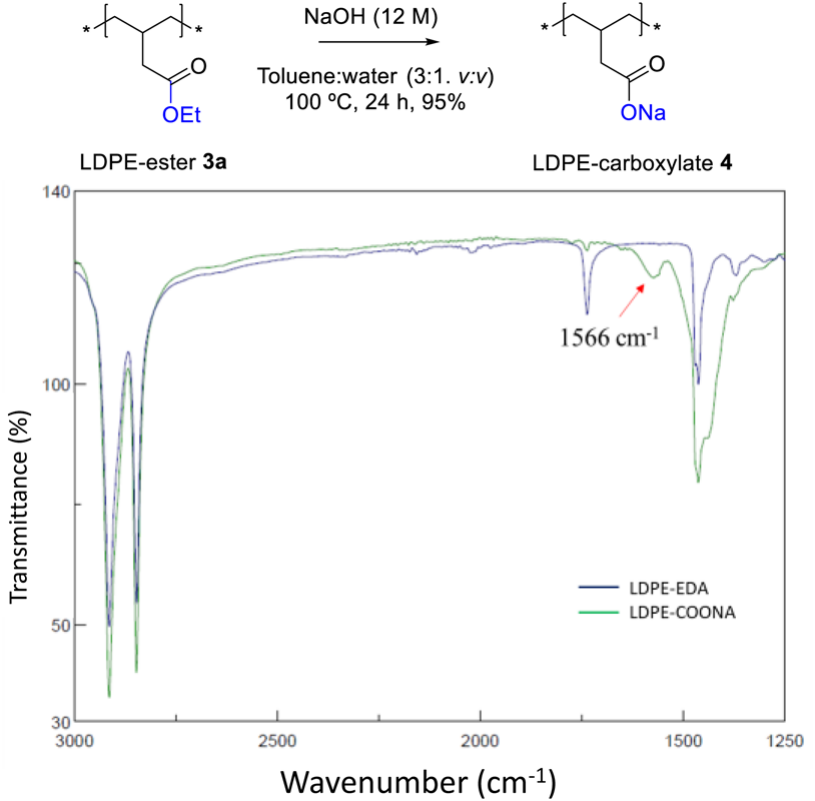
FT-IR spectra of LDPE-ester 3a (LDPE-EDA) before and after treatment with a 12 M aqueous NaOH solution in toluene (3 : 1 v : v) at 100 °C for 24 h, to give LDPE-carboxylate 4 (LDPE-COONa).

Fig. 4 shows the FT-IR spectrum of LDPE-ester 3a treated with boiling NaOH (in water or ethanol) or boiling concentrated HCl, during 1 h reaction time. LDPE-ester 3a does not change and is recovered, unmodified, after the different treatments. These results indicate than LDPE-ester 3a is stable towards hydrolysis in water at extreme pHs (0–14), due to the high hydrophobicity of the very long alkyl chain.^14^ However, Fig. 5 shows that if toluene is added to the treatment with aqueous NaOH (3 : 1 v : v) and the reaction time is longed for 24–48 h, the corresponding LDPE-carboxylate 4 is obtained, as assessed by the new peak at 1566 cm^−1^ in FT-IR, which can be assigned to Na^+^ carboxylate bound to PE (Table S1†). The conversion of the reaction can be calculated by the decrease in intensity of the peak of the ethyl ester group in 3a (1735 cm^−1^), and is here >95%. The hydrolysis reaction can also be carried out successfully with 2-methyltetrathydrofurane as a solvent instead of toluene (Fig. S7†), the former being a recognized sustainable solvent from biomass.^15^ Nevertheless, it is worthy to comment here that toluene is one of the easiest organic solvents to be recycled and separated from water (*i.e.* by distillation and cooling), and that will be employed during our study here in the minimum amount possible to treat the polyethylene material.

### 2.3 Failed trans-esterification and trans-amidation reactions of LDPE-ester 3a

With gram amounts of the PE-ester 3a in hand, we tested a further functionalization of the polymer by trans-esterification and trans-amidation reactions.^16^ It is worthy to comment here that we used industrial esterification methodologies (Brønsted acid- or base-catalyzed procedures) and avoided other techniques common in the laboratory but hardly scalable at multikilogram amounts (such as activation with carbodiimides). Nevertheless, PE-ester 3a showed reluctant to transesterification reactions. First, we used a 4 M and 12 M HCl_aq._ solutions for the transesterification reaction of PE-ester 3a with oleyl and 2-ethyl hexyl alcohol as reactants, however, the FT-IR signals in LDPE-ester 3a remained unchanged (Fig. S8†). It is true that the ester signal could coincide for the new esters, however, the fact that any other new signals of the alcohol molecules appear in the FT-IR spectra (*i.e.* the alkene signals for oleyl alcohol) and that PE-ester 3a is stable under such acid reaction conditions (see Fig. 4 above) support that the trans-esterification reaction has not occurred and the desired products PE-esters 5–6 have not been formed. When we switched to basic conditions (4 M and 12 M NaOH_aq._), the only product found was the white solid LDPE-carboxylate 4 (the new peak at 1642 cm^−1^ corresponds to hydrogen bridging in the aqueous media, since the sample is not dry, Fig. S9†).

Trans-amidation reactions with oleylamine and 2-ethyl hexyl amine were then tested. The same disappointing results were found in acid media, only slightly observing the fingerprint amide bands at 1609 cm^−1^ and 1485 cm^−1^ for oleylamine when a 4 M HCl solution was employed (Fig. S10†). The desired PE-amides 7–8 were found only in minor amounts and the use of 12 M HCl led, in this case, to some hydrolysis to get LDPE-carboxylic acid 9. These results reflect the tendency of amines to protonate, faster than alcohols. Under basic conditions, the main product was LDPE-carboxylate 4, but some transamidation reaction with oleylamine to PE-amide 7 could be observed with a 4 M NaOH solution (Fig. S11†).

These failed trans-esterification and trans-amidation reactions can be interpreted on the basis of the stability of LDPE-ester 3a towards hydrolysis, thus also towards transesterification and transamidation reactions. We then attempted to achieve the new trans-esterified and trans-amidated materials from LDPE-carboxylate 4, which has already suffered the nucleophilic attack and could be ready for further transformations on the carbonyl group.

### 2.4 Trans-esterification and trans-amidation reactions of LDPE-carboxylate 4

**Fig. 6:**
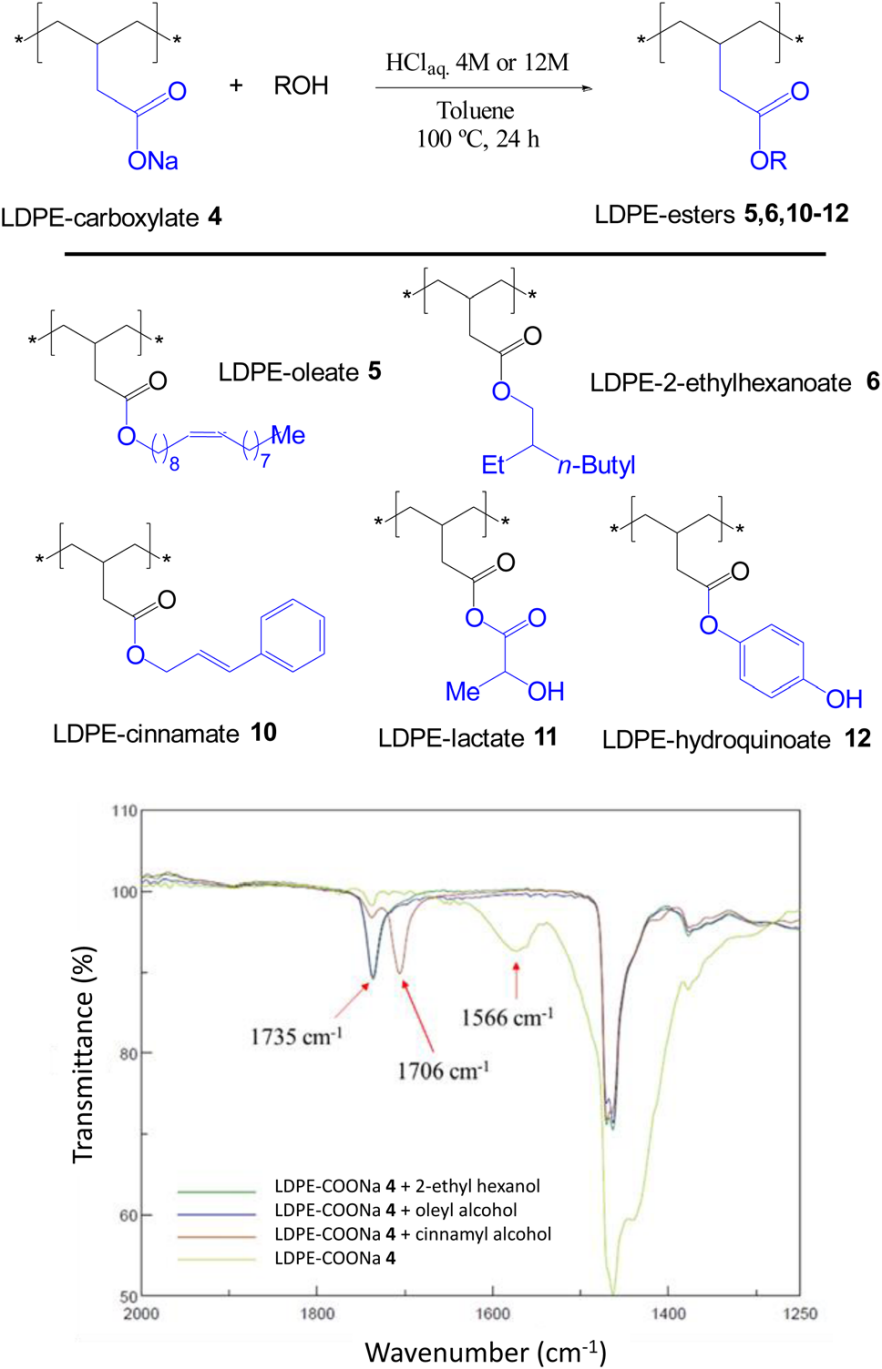
New LDPE-esters 5, 6, 10–12 prepared by transesterification reaction of LDPE-carboxylate 4 (LDPE-COONa) with different alcohols under acid conditions, and the corresponding FT-IR spectra of 4–6 and 10. Yields are quantitative for all the new materials except for 10, which contains a significant amount of LDPE-carboxylic acid 9 (see also Fig. S12 and S13†).

**Fig. 7:**
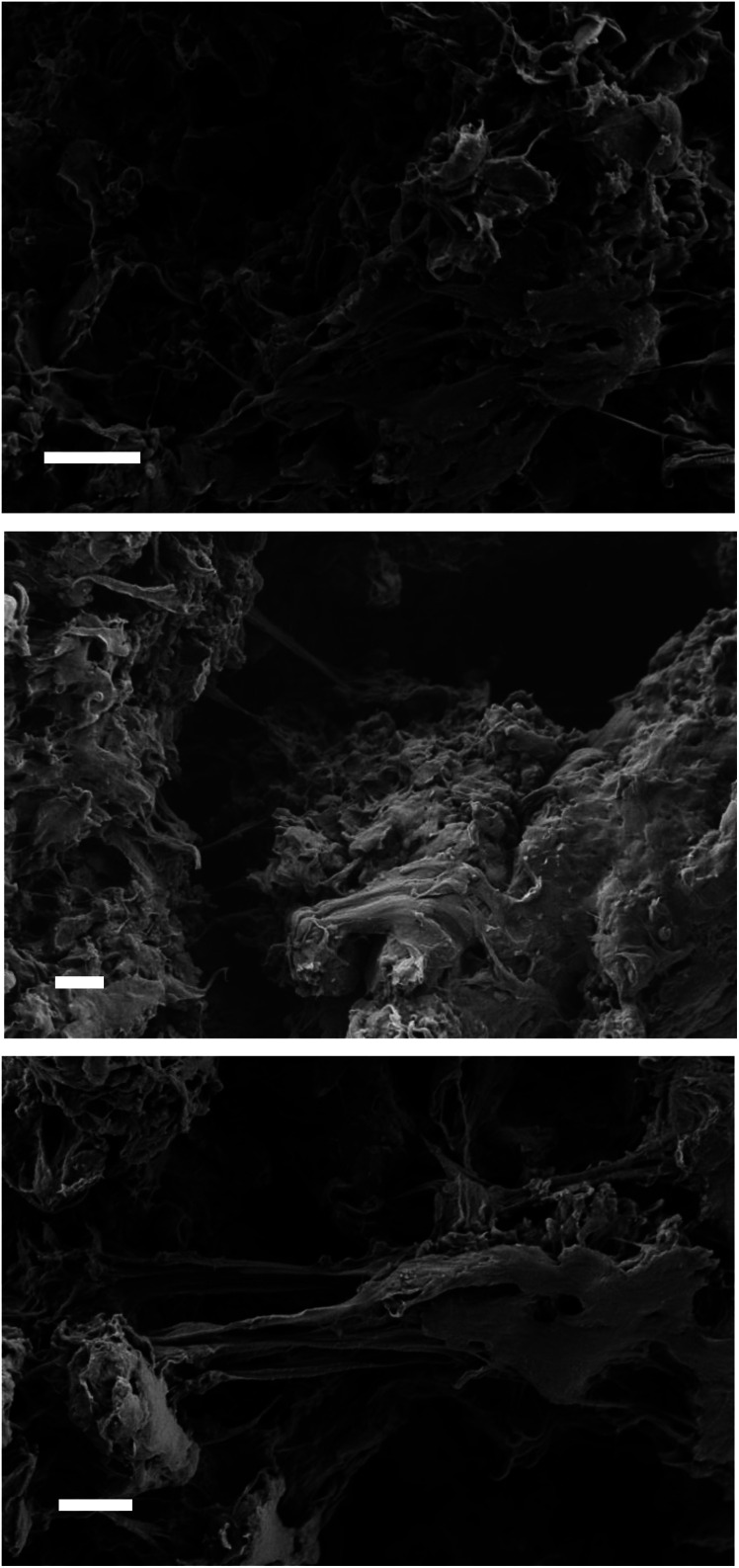
SEM images of LDPE (top), LDPE-oleate 5 and LDPE-2-ethyl hexylate 6. White bars in each image correspond to 2 μm.

The acid-catalyzed trans-esterification reaction of LDPE-carboxylate 4 was performed with different alcohols and the results are shown in Fig. 6. It can be seen that five different alcohols of different nature are now incorporated into the polymer in quantitative yields, according to the appearance of the ester signal and the complete loss of the carboxylate signal at 1566 cm^−1^ in the FT-IR spectra, and the mass recovered after work-up. Please notice that the FT-IR signals of the new LDPE-esters differ significantly of LDPE-ester 3a and contains the rest of signals expected for the covalently anchored alcohols (Fig. S12 and S13†). Only LDPE-cinnamate 10 showed significant amounts of carboxylic acid 9. Fig. 7 shows the SEM images of the trans-esterified LDPE materials 5 and 6, showing that the microstructure of the polymer remains preserved after the synthetic steps. The reactions could also be carried out successfully, with similar results, with 2-methyltetrathydrofurane as a solvent instead of toluene. The hydrolysis tests for the new LDPE-esters 5 and 6 show that both polymers resist the hydrolysis in both acid and basic media, even at 100 °C for 24 h reaction time (Fig. S14 and S15†). These results indicate that a variety of hydrolytically-stable PE esters can be synthesized with the methodology described here.

The acid-catalyzed trans-amidation reaction was also attempted under different conditions, however, the corresponding LDPE-amides were not found, but only LDPE-carboxylic acid 9 (Fig. S16†). These results can again be explained by the higher basicity of the amines, which will be protonated under the reaction conditions employed.

### 2.5 Functionalization of PE with maleic anhydride (MA)

**Fig. 8:**
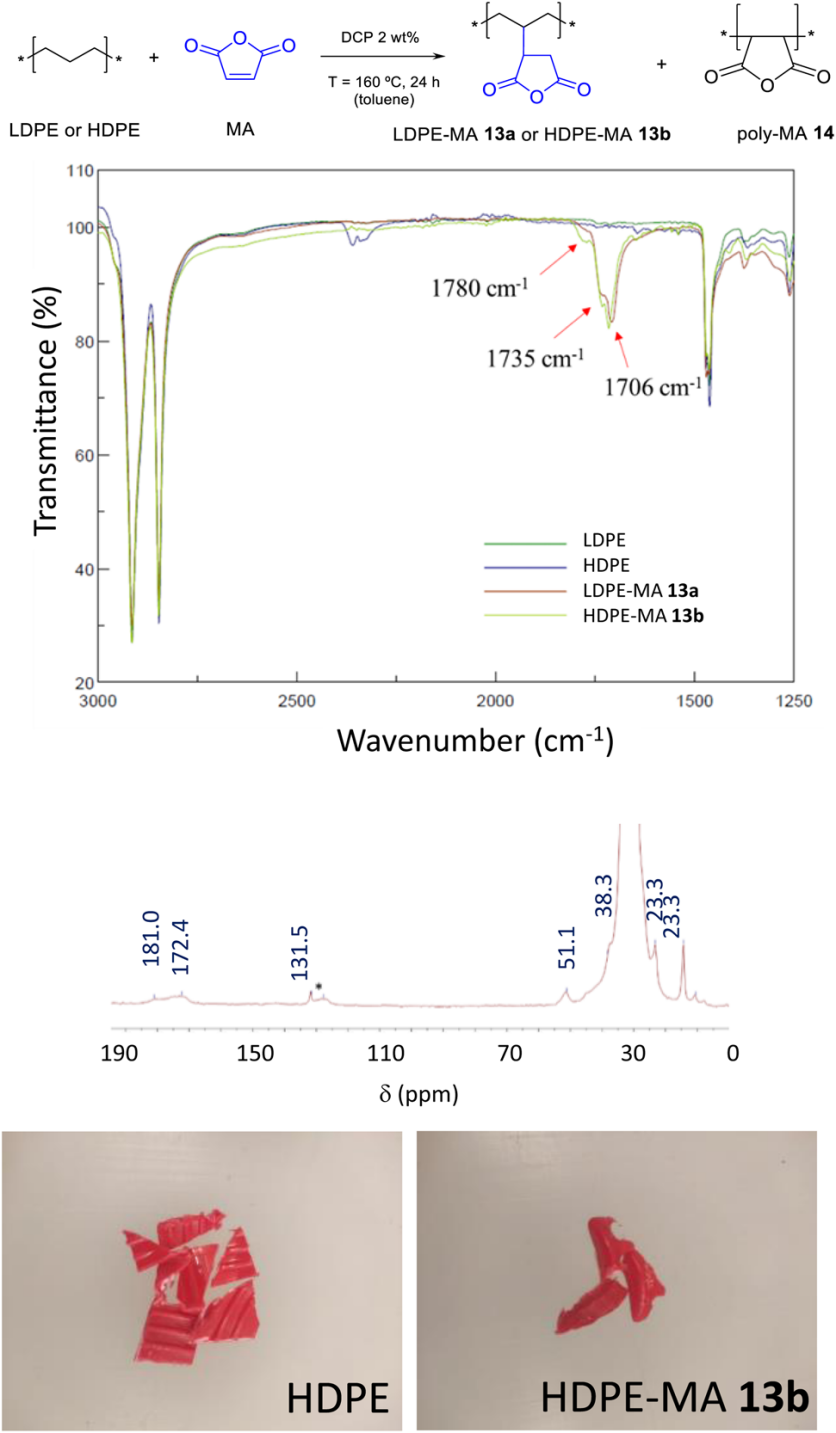
Functionalization of LDPE and HDPE with maleic anhydride (MA) under radical conditions. (Top) Reaction conditions (DCP: dicumyl peroxide). (Middle top) FT-IR spectra of the new materials. Middle bottom: ^13^C SS-MAS-NMR spectrum of LDPE-MA 13a [the signal at 131.0 ppm (*) is tentatively assigned to *in situ* formed PE alkenes]. (Bottom) Photographs of HDPE before and after incorporation of MA (HDPE-MA 13b).

The incorporation of maleic anhydride to PE was attempted under reported reaction conditions, using a 1 wt% of dicumyl peroxide (DCP) as a radical initiator at 160 °C, a reaction temperature where LDPE is molten (Fig. S17†).^5^ However, in our hands, the loading of MA incorporated was lower than 0.5 wt% (Fig. S18 and S19†). Thus, we explored the use of toluene as a solvent, which is able to dissolve LDPE and has shown success in the ester functionalization (see above). Fig. 8 shows that, indeed, both LDPE and HDPE are functionalized with MA in toluene at 100 °C when a 2 wt% of the DCP radical catalyst is employed (see also Fig. S19†). The loading of ester in the polymer is as high as 7.0 wt% and 7.4 wt% for LDPE-MA 13a and HDPE-MA 13b (in the form of cut red bottle caps), respectively, according to EA and FT-IR measurements, when mesitylene is used as a solvent at 130 °C. In average, the new materials contain 1/78 functionalized CH_2_ units. It has to be noted here that, as it occurs for the functionalization with EDA, the washing procedure is key to achieve the desired MA-functionalized PE, since poly-maleic anhydride 14 (polyMA) is also formed during the radical reaction (Fig. S20†).^18^ Thus, the resulting solid material after MA functionalization was washed with hot water (50 °C) to selectively dissolve polyMA 14, whose removal was assessed by the disappearance of the FT-IR peaks at 1856 cm^−1^ and 1780 cm^−1^, associated to the asymmetric and symmetric stretching vibrations of poly-MA 14, respectively (Fig. S21†). The FT-IR spectra of the washed materials show, beyond the peaks correspond to PE-MA appearing at 1780 cm^−1^ and 1735 cm^−1^, a new peak at 1706 cm^−1^ which corresponds to partially hydrolyzed MA (with a carboxylic acid, see Fig. 8). The ^13^C SS-MAS-NMR of LDPE-MA 13a is also shown in Fig. 8, and the two desymmetrized carbonyl signals of the covalently anchored anhydride can be seen at 181.0 and 172.4 ppm. The key signal of the new CH formed, which bonds PE and MA, clearly appears at 51.1 ppm, as well as the other expected CH_2_ and CH_3_ signals of the new polymer. Please notice here that LDPE-MA 13a is not soluble in any solvent tested due to the inter-crossing of the PE chain during the radical reaction, thus liquid phase NMR could not be performed (Fig. S22†).

### 2.6 Scaling-up and comparative characterization of the physicochemical and mechanical properties for LDPE-MA 13a, pristine LDPE and re-precipitated LDPE

**Fig. 9:**
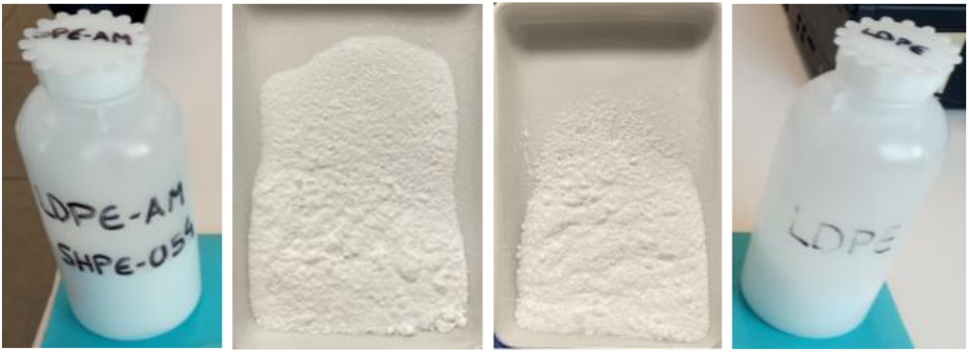
Photographs of 100 hundred grams of LDPE-MA 13a (left) and precipitated LDPE (right).

The synthesis of LDPE-MA 13a could be scaled up to prepare a hundred grams of solid polymer. Fig. 9 shows a photograph of the new material. For the sake of comparison, pristine LDPE was re-precipitated after dissolving and being treated under the same reaction conditions than 13a but without any other reactant, and the corresponding aspect of the re-precipitated material is also shown in Fig. 9. This last material was prepared and characterized to completely discard any influence of the dissolving-precipitating steps on the polymer's properties.

These polymers, together with pristine LDPE, were characterized by gel permeation chromatography (GPC), contact angle and surface tension measurements, dynamic mechanical analysis (DMA), X-ray diffraction, thermogravimetric analysis (TGA) and differential scanning calorimetry (DSC).

#### 2.6.1 Gel permeation chromatography

**Fig. 10:**
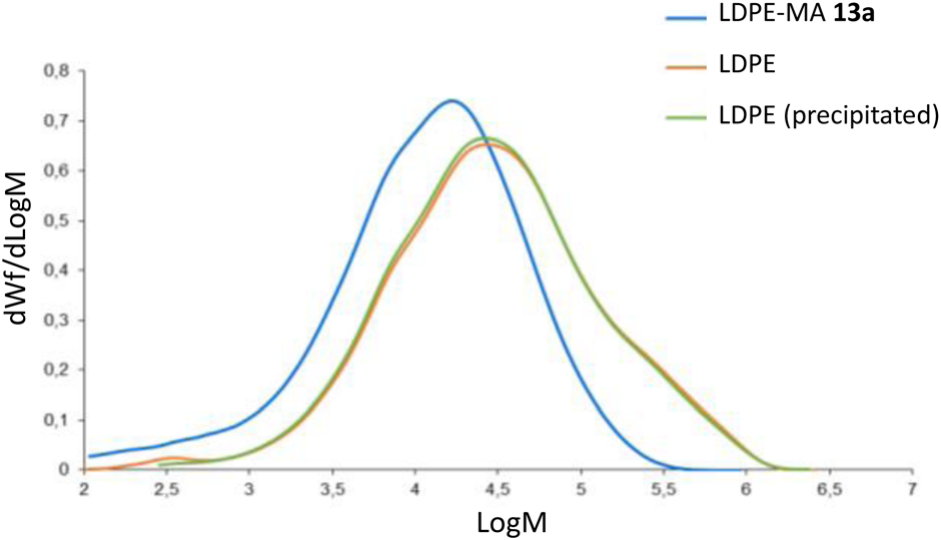
GPC chromatograms for LDPE-MA 13a (blue), commercially available LDPE (orange) and precipitated LDPE (green).

Fig. 10 shows the plot GPC chromatograms for the commercially available LDPE, precipitated LDPE and LDPE-MA 13a. An overlay of the molar mass distributions is represented, and Table 1 shows the numeric results. According to molar mass averages and molar mass distributions, LDPE-MA 13a shows a lower weight-average molecular weight (*M*_w_), with a shift in the molecular weight distribution compared with original and precipitated LDPE. The slight decrease in the molecular weight of the precipitated sample respect to pristine LDPE was expected according to the literature.^1*d*,19^ However, LDPE-MA 13a suffers a displacement of the position of the dominant fractions towards much lighter molecular masses, and this result is supported by the shape of the distribution curve, where the lower content of heavier compounds compared to pristine LDPE and precipitated LDPE can be clearly seen. In contrast, LDPE-MA 13a shows a much lower polydispersity index than the original LDPE samples (*M*_w_/*M*_n_ = 7 *vs.* 9–10), which enables a higher SEM images of LDPE-MA 13a confirm the higher density of the inter-crossed LDPE-MA 13a material compared to pristine LDPE (Fig. S23†). These results may be caused by some fractionation during the precipitation process, as previously reported.^1*d*^

**Table 1:** Numeric results for the GPC experiments on LDPE-MA 13a, commercially available LDPE and precipitated LDPE. The weight average molecular weight (*M*_w_) and number-average molar mass (*M*_n_) has been calculated using between polystyrene standards (see experimental). The IVcc value has been calculated from *M*_v_ and Mark Houwink coefficients, from a polyethylene sample

	LDPE-MA 13a	Commercial LDPE	Precipitated LDPE
*M*_w_ (g mol^−1^)	24 600	82 000	79 000
*M*_n_ (g mol^−1^)	3400	8300	10 600
*M*_w_/*M*_n_	7.1	9.9	7.4
*M*_z_ (g mol^−1^)	71 200	352 300	343 400
*M*_p_ (g mol^−1^)	16 700	27 300	26 400
*M*_v_ (g mol^−1^)	20 500	63 400	61 100
IVcc (dL g^−1^)	0.59	1.34	1.3

#### 2.6.2 Contact angle and surface tension

Contact angle is a visible measure of the wettability process. It depends not only on the surface tension of the solid and liquid but also on the contribution of intermolecular interactions, dominated by short-range interactions such as van der Waals, dipolar and hydrogen bonding interactions.^20^ The contact angle value is particularly informative for the structurally heterogeneous, “non ideal” solid LDPE, in which the interfacial functional groups are disordered in both position and orientation. The influence of molecular weight (*M*_n_), molecular weight dispersity (MWD) and organic functional group present at the solid–liquid interface was evaluated using the pendant drop method, with the surface tension of water and iodomethane over the different LDPE samples. Besides, the kind of functionalization surface depends on its thickness and the orientation of functionalized molecules in this layer. Then, since the modified LDPE material changes the hydrophobic and hydrophilic balance of the surface, compared to the pristine one, a relationship between the wettability of the surface-functionalized LDPE sample and the structure of the organic functional group present at the solid–liquid interface can be determined by the contact angle.^21^ If the molecules of the modified layer are oriented by the hydrophilic groups toward the air phase, hydrophilicity of the solid surface should increase and correspond to the hydrophilic properties of a given molecule in this surface.

The measurements of the contact angle at 20 °C for the different LDPE samples with an apolar (diiodomethane) and a polar (water) liquid (see Fig. S24 and S25†) are summarized in Table 2. The calculated values of surface tension are in good agreement to those reported in the literature.^22^

**Table 2:** Measured values (by triplicate) of contact angle with distilled water (*θ*_w_) and diiodomethane (*θ*_D_) for LDPE-MA 13a, commercially available LDPE and precipitated LDPE samples

LDPE sample	*θ*_w_	*θ*_D_
LDPE-MA 13a	86.34	53.82
	89.73	50.25
	89.22	51.30
Commercial LDPE	94.45	64.94
	85.36	57.07
	97.72	61.01
Precipitated LDPE	91.96	60.06
	81.90	60.12
	91.10	68.60

As expected, the LDPE original samples showed a high contact angle, approximately 93° in water. LDPE-MA 13a showed a somewhat lower but still relatively high contact angle value of 88°. This contact angle data provides physical evidence of the surface modification imparted by the incorporated ester groups, as previously described,^23^ which is in good accordance with the enhanced hydrolytic stability of the material.

The hydrolytic stability of LDPE-MA 13a must also be related to the surface tension. The latter practically does not depend on temperature but mainly in Lifshitz–van der Waals intermolecular interactions and polymer molecular weight. In principle, the surface tension increases with molecular weight, however, the influence of the molecular weight on the surface tension value decreases significantly when the molecular weight exceeds certain molecular weight (41 200 g mol^−1^ in the case of polystyrene sample), which is attributed to thermal degradation.^22*a*^ Since the average molecular weight of LDPE-MA 13a is significantly lower than pristine LDPE, but van der Waals intermolecular interactions are expected to be much higher due to the presence of the ester groups, we measured the surface tension values, which are depicted in Table 3.

**Table 3:** Surface tension values (by duplicate) for LDPE-MA 13a, commercially available LDPE and precipitated LDPE samples

LDPE sample	*γ* (mN m^−1^)
LDPE-MA 13a	30.71 ± 5.20
	30.00 ± 3.92
Commercial LDPE	27.69 ± 0
	28.96 ± 2.71
Precipitated LDPE	32.48 ± 0
	31.71 ± 0

The results show the surface tension of LDPE-MA 13a is at least equal if now higher than pristine LDPE, despite having a much lower molecular weight. These results further support the enhanced hydrolytic stability of the new material. Notice that the total surface tension of any PE is considerably lower using the contact angle of strong polar liquid as a measurement, instead that the contact angle of apolar liquids.^22*c*^

#### 2.6.3 Dynamic mechanical analysis

**Fig. 11:**
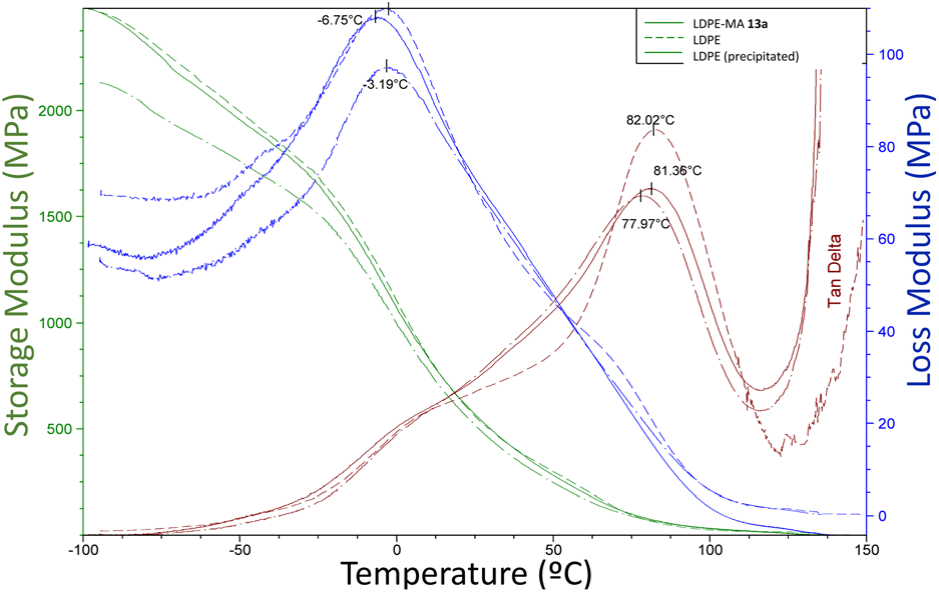
Variations of mechanical *E*′, *E*′′ and tan *δ* with temperature for the different LDPE samples.

The dynamic mechanical analysis (DMA) provides simultaneous observations of the storage modulus *E*′, loss modulus *E*′′ and damping factor (tanget tan *δ*), which are plotted as functions for temperature (−100 to 150 °C) in Fig. 11. These values give information about the structural deformation mechanism in the material associated with thermal response.

The storage modulus for all the LDPE samples (green lines, *E*′), including LDPE-MA 13a, is very similar, indicating similar crystallinity and low changes in chains rigidity. The tan *δ* curves (red lines) give two informative peaks, named α and β relaxation peaks. The α relaxation value is attributed to the vibrational and reorientation motion within the crystals or even, as later was described,^24^ the relaxation of the constrained molecules with reduced mobility located near the crystallites. The β relaxation corresponds to the glass transition of the amorphous regions, and the temperature is assigned to *T*_g_. If we analyse the results, the peaks for β relaxation are negligible, however, the temperature for the α peaks, also shown in Fig. 11, are similar for the three LPDE samples when subjected to oscillatory strain, all around 78–82 °C, typical for LDPE samples with high crystallinity.^25^ In other words, LDPE-MA 13a presents a similar dynamic mechanical analysis values than the original counterparts.

#### 2.6.4 Thermogravimetric analysis (TGA), differential scanning calorimetry (DSC) and X-ray diffraction (XRD) analysis

**Fig. 12:**
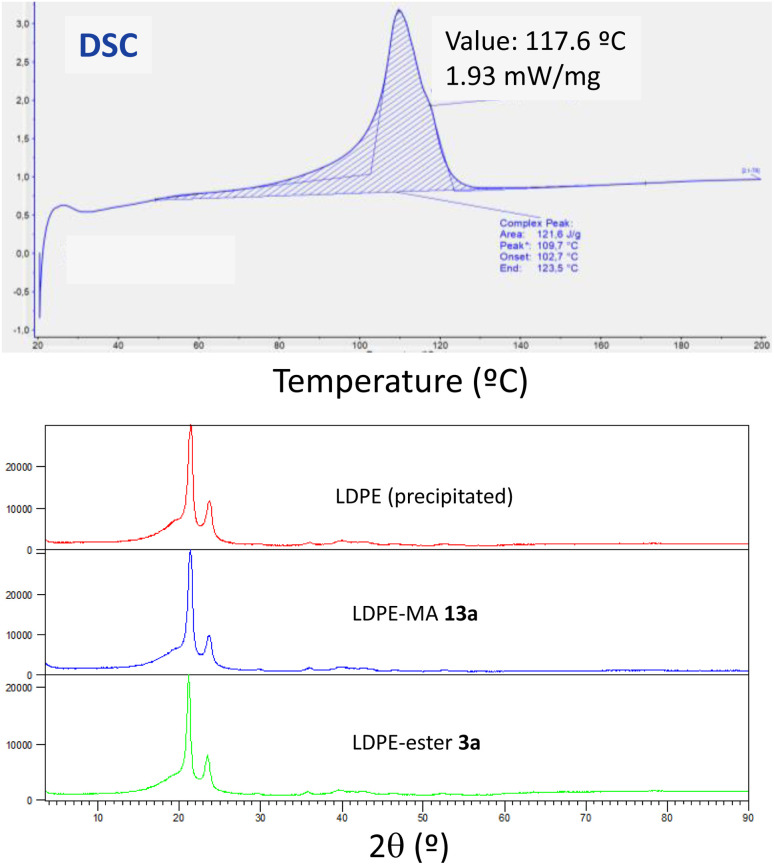
(Top) Differential scanning calorimetry (DSC) for LDPE-MA 13a, first heating (for second heating see Fig. S29†). (Bottom) X-ray diffractograms (XRD) for precipitated LDPE, LDPE-MA 13a and, for the sake of comparison, LDPE-ester 3a.

The thermogravimetric analysis for the three LDPE samples shows again very similar values, with the onset of decomposition occurred at temperature around 462–467 °C (Fig. S26–S28†). It is noteworthy that the higher temperature corresponds to LDPE-MA 13a, indicating the good thermal stability of this sample despite the chemical modification. This slight improvement in thermal stability for LDPE-MA 13a was confirmed by differential scanning calorimetry (DSC). Fig. 12 (top) shows that the melting temperature for LDPE-MA 13a is 109.7. The polymer started to lose its solid form at around 70 °C, which corresponds to the glass transition temperature for this sample. Fully crystalline polymers do not have any glass transition, and their structure remains intact until the melting point. For this reason, the LDPE-MA 13a sample must be considered as a semicrystalline polymer. X-ray diffraction (XRD) measurements confirmed the crystallinity of the new material and the similarity with pristine LDPE (and also with LDPE-ester 13a), as also shown in Fig. 12 (bottom).

The onset melting point for LDPE-MA 13a appears at 102.7 °C as an endothermic process and, as the temperature increases, the rate of heat flow also rises until reaching to the melting point at 109.7 °C. Then, the process started to release energy dramatically until the end point at 123.5 °C. The second cycle presents the peak point at 108.6 °C (Fig. S29†). If we compare these results with pristine LDPE (Fig. S30†), the thermal stability of LDPE-MA 13a is confirmed. The melting temperature for virgin LDPE sample is 115.6 and the glass transition temperature for this sample is around 83 °C, with the onset melting point appearing at 104.7 °C. These values are in concordance with previously LDPE samples reported^26^ and are just +5 °C compared to the new LDPE-MA 13a sample, despite the esterification suffered by the polymer structure, which further supports the stability of the new material.

### 2.7 Hydrolysis of LDPE-MA 13a

**Fig. 13:**
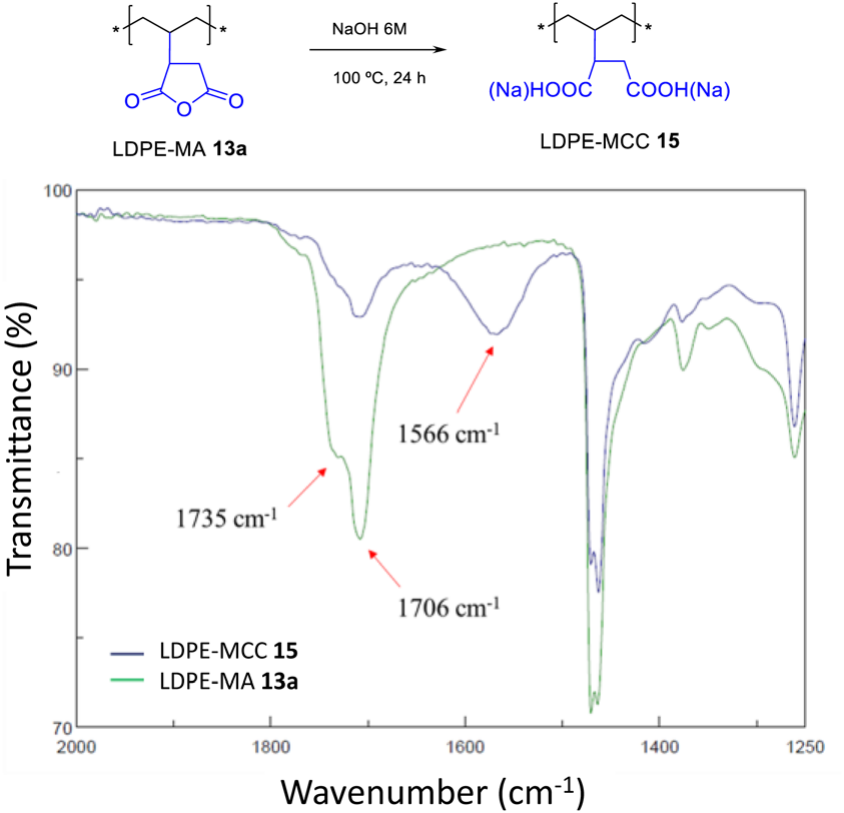
Hydrolysis of LDPE-MA 13a under the indicated basic conditions. The FT-IR spectra show the disappearance of the MA signals together with the appearance of the signals for both the carboxylic acid and the carboxylate.

LDPE-MA 13a was submitted to hydrolysis with HCl and NaOH at room temperature, showing no signs of degradation after 24 h (Fig. S31†). However, Fig. 13 shows that the hydrolysis of LDPE-MA 13a occurs in boiling NaOH_aq._ 6 M after 24 h. It can be seen that the anchored MA cycle indeed opens to a mixture of carboxylic acid and carboxylate (MCC), to give LDPE-MCC 15.^17^

### 2.8 Trans-esterification and trans-amidation reactions of LDPE-MA 13a

**Fig. 14:**
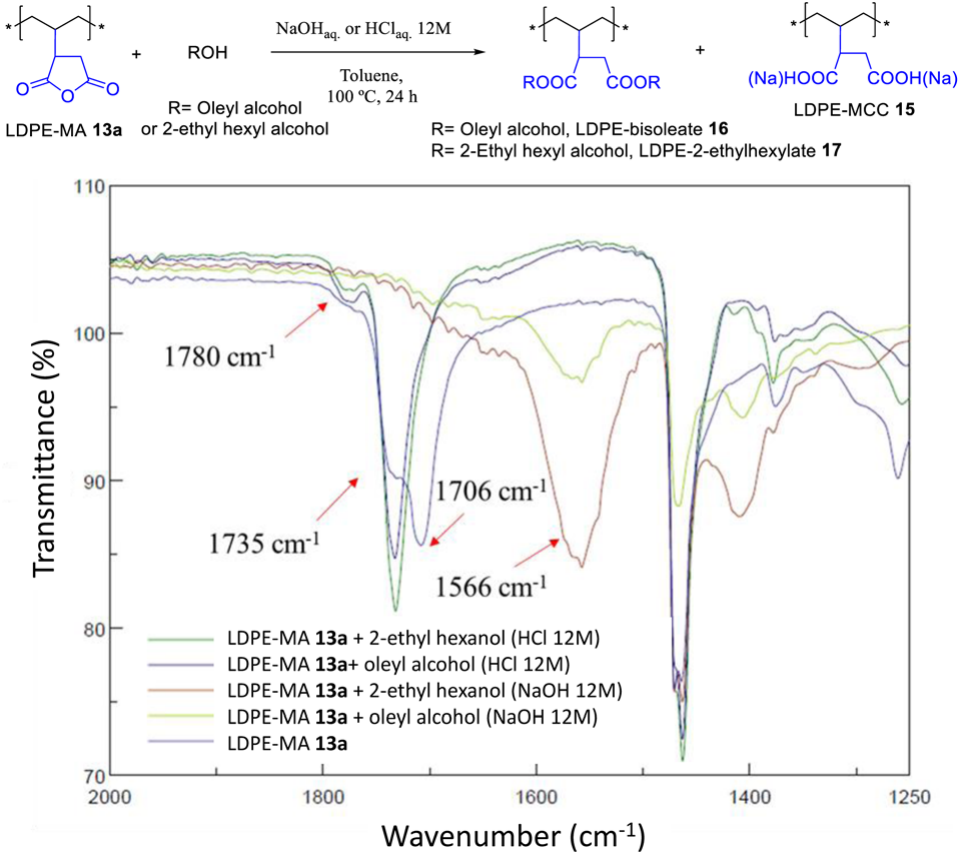
Trans-esterification reactions of LDPE-MA 13a under the indicated basic or acid conditions. The (FT-IR) spectra show the formation of the diesters under acid-catalyzed conditions.

The acid-catalyzed trans-esterification reaction of LDPE-MA 13a with both oleyl and 2-ethyl hexyl alcohol gave the diester-PE materials LDPE-bisoleate 16 and LDPE-bis-2-ethylhexanoate 17 in good yields according to the recovered mass. Fig. 14 shows that the new signal of the ester in FT-IR, at 1735 cm^−1^, is the only signal found together with the original LDPE signals, and that the signals corresponding to MA have completely disappeared. In contrast, the reaction under basic conditions yielded the hydrolysis product LDPE-MCC 15. The hydrolysis test for LDPE-bisoleate 16 confirms the resistance of the material to both acid and base conditions (Fig. S32†).

**Fig. 15:**
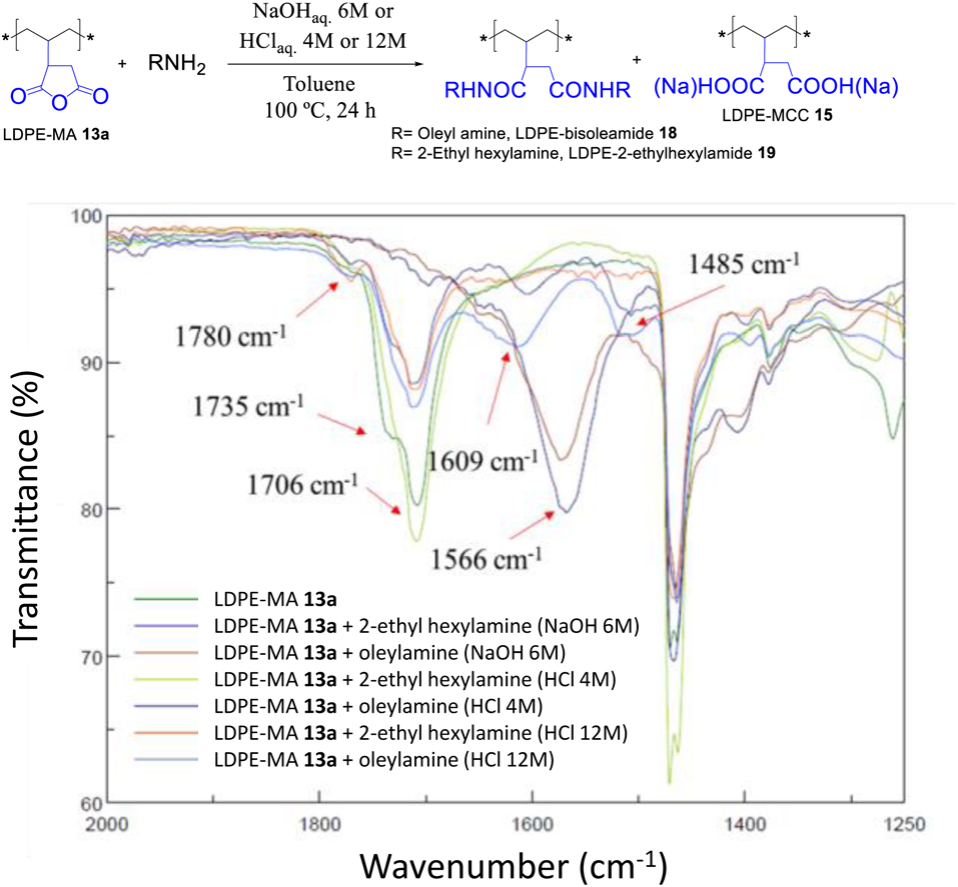
Trans-amidation reactions of LDPE-MA 13a under the indicated acid or basic conditions. The FT-IR spectra show the formation of bisamide LDPE-oleylamide 18 under acid-catalyzed conditions.

The trans-amidation reaction of LDPE-13a with oleyl and 2-ethylhexylamine was attempted under similar reaction conditions. Although the later did not work, the former coupled under acid-catalyzed conditions, to give the desired LDPE-bisoleylamide 18 in good yield according to the recovered weight and the FT-IR signals in Fig. 15. The hydrolysis test of LDPE-bisoleylamide 18 showed no signs of decomposition under acid or base reaction media (Fig. S33†).

### 2.9 Functionalization of PE with acrylates

**Fig. 16:**
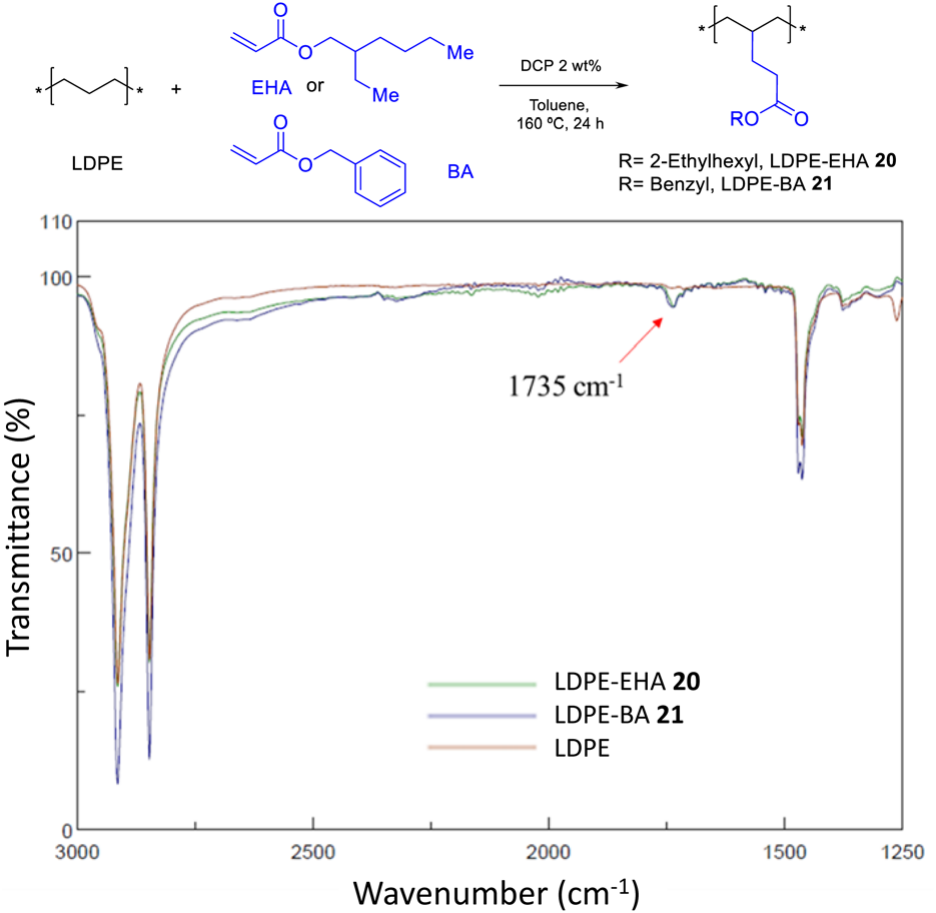
Functionalization of LDPE with acrylates. The FT-IR spectra show the characteristic ester signal at 1735 cm^−1^.

The incorporation of EDA allows to get monoesters covalently anchored on the PE structure, and the incorporation of MA yields diesters. In order to get also monoesters by the radical catalyzed procedure, the bonding of 2-ethyl hexyl acrylate (EHA) and benzyl acrylate (BA) was attempted under the same optimized reaction conditions found for MA. Fig. 16 shows that, indeed, the acrylates are incorporated in LDPE, although still in low yields (<10%). Further studies may drive to higher loadings. Hydrolysis tests for LDPE-EHA 20 showed the resistance of the material to acid and base conditions (Fig. S34†).

## 3 Conclusions

Hydrolytically stable LDPE and recycled HDPE with pending ester, amide, carboxylic acid and carboxylate groups have been synthesized. The solid materials have, in average, 1/78–120 functionalized CH_2_ units and are stable to hydrolysis under extreme pH conditions (0–14). These results open the way to upcycle PE with a variety of new functionalities^27^ preserving and taking advantage the original PE backbone.^28^ While ester functionalization of PE is not new, our study here significantly changes the perspective and relevancy of this synthetic approach, focusing on tackling the environmental challenge now posed by polyethylene waste. In this regard, three necessary advances towards that objective are made: scaling-up, high ester functionalization (up to a 7 wt% compared to just 1 wt% before) and functionality of the upcycled material (hydrolytically stable). Future work should include data regarding the ability of the materials to act as a lubricant when in contact with water, for instance about how it affects wear of a test component when compared to typical ester lubricants or how effective it is at remaining separate from water over long periods of time.

## Author contributions

S. H.-A. carried out most of the reactions, and found the solvent-mediated functionalization with MA and acrylates. B. P-dL found and characterized the functionalized ester PE material (also recycled) and carried out the transesterification and transamidation reactions. C. B. performed further PE functionalization and characterization experiments, including hydrolysis. P. M.-V. carried out hydrolytic experiments. M. V. designed and interpreted the comparative physicochemical and mechanical characterization of LDPE materials. J. O.-M. supervised the project and wrote the manuscript. A. L.-P. conceived the idea, supervised the whole project and wrote the manuscript. All authors have contributed and agree with the final version of the manuscript.

## Conflicts of interest

A related patent co-authored by A. L.-P., S. H.-A. and J. O.-M., with application number P202230680, has been submitted in Spain.

## Supplementary Material

RA-013-D3RA05024F-s001
