# Supplementary material for: Functionalization of polyethylene with hydrolytically-stable ester groups
Source: RSC Adv. 2023 Aug 10;13(34):23859–69. doi: 10.1039/d3ra05024f (PMC10413336; doi:10.1039/d3ra05024f)
Supplement: RA-013-D3RA05024F-s001 [file RA-013-D3RA05024F-s001.pdf]

Electronic Supplementary Material (ESI) for RSC Advances.
This journal is © The Royal Society of Chemistry 2023

Electronic Supplementary Information

Functionalization of polyethylene with hydrolytically-stable ester groups

Susi Hervàs-Arnandis,[a] Brenda Palomar-de Lucas,[a] Cristina Bilanin,[a] Paloma Mingueza-Verdejo,[a] Mónica Viciano,[b] Judit-Oliver Meseguer*[a] and Antonio Leyva-Pérez.*[a]

[a] Instituto de Tecnología Química (UPV–CSIC), Universidad Politècnica de València–Consejo Superior de Investigaciones Científicas, Avda. de los Naranjos s/n, 46022 Valencia, Spain.

[b] AIMPLAS, València Parc Tecnològic, C/ Gustave Eiffel, 4, 46980 Paterna, Valencia, Spain.

Corresponding authors´ e–mails: joliverm@itq.upv.es, anleyva@itq.upv.es.

Phone: +34963877812; Fax: +349638 77809.

Table of Contents

1 Experimental Section……………………………………………………………….…ESI2

1.1 Materials………………………………………………………………..…………ESI2

1.2 Characterization techniques…………………………………………………..…ESI2

1.3 Reaction procedures and product characterization……………………………...........ESI3

2 Tables S1-S2………………………………………………………………...…..…ESI9

3 Figures S1-S34…………………………………………………………………......ESI10

1 Experimental Section.

1.1 Materials.

All reagents and solvents were purchased from commercial sources and used as received otherwise specified. The following reagents were obtained from Sigma Aldrich: Low-density polyethylene (LDPE), melt index: 25 g/10 min (190 ºC/2.16 kg), melting point: 116 ºC, density: 0.925 g/ mL in granule form, CAS: 9002-88-4; Maleic anhydride (AM), Mr = 98.09 g/mol, solid, melting point: 51-53 ºC, boiling point: 202 ºC/1013 hPa, CAS: 108 31 6; dicumyl peroxide (DCP), Mr = 270.37 g/mol, solid, melting point: 39-41 ºC, density 1.56 g/mL at 25 ºC, CAS: 80-43-3; ethyl diazoacetate (EDA), 15% in toluene, Mr = 114.10 g/mol, liquid, density 0.895 g/mL at 25 ºC, boiling point: 141 ºC (95.99kPa), CAS: 623- 73-4; 2-ethyl-1-hexanol, Mr = 130.23 g/mol, liquid, boiling point: 183-186 ºC, CAS: 104-76-7; oleyl alcohol, Mr = 268.478 g/mol, liquid, density: 0.849 g/mL at 25 ºC, boiling point: 207 ºC/ 13 mmHg, CAS: 143-28 2; cinnamyl alcohol, Mr = 88.148 g/mol, liquid, boiling point: 250 ºC, density: 1.044 g/mL, CAS: 104-54-1; 2-ethylhexylamine, Mr = 129.247 g/mol, liquid, density: 0.789 g/mL at 25 ºC, boiling point: 169 ºC, CAS: 104-75 6; oleylamine, Mr = 267.493 g/mol, liquid, boiling point: 348-350 ºC, CAS: 112-90-3; 2 ethylhexyl acrylate, Mr= 184.27 g/mol, liquid, boiling point: 215-219 ºC, CAS: 103-11-7; benzyl acrylate, Mr = 162.19 g/mol, liquid, CAS: 2495-35-4. Recycled HDPE was obtained from the cap of a used water bottle.

1.2 Characterization techniques.

Elementary analysis: The carbon, hydrogen, nitrogen and sulfur content of the polyethylene samples functionalized with MA or EDA were quantified with a Fisons EA 1108CHN-S analyzer.

Fourier Transformed Infrared (FT-IR) Spectroscopy: Attenuated total reflectance (ATR) Fourier transform infrared (FTIR) measurements of solid and liquid samples were recorded with 50 scans from 400 to 4000 cm^{-1} on a JASCO FT/IR 4700 series spectrophotometer.

Field emission scanning electron microscopy (SEM): The sample was supported on a grid and measured with a ZEISS Ultra-55 instrument, from Oxford Instruments.

Nuclear magnetic resonance (NMR): Liquid ^{1}H, ^{13}C and DEPT measurements were recorded on a 400 MHz Bruker Avance instrument at room temperature, 70 or 95 ºC using deuterated toluene as a solvent, containing TMS as internal standard. Solid state, magic angle spinning (SS-MAS) ^{13}C NMR measurements of the solids were recorded with a 400 MHz Bruker Oxford-Avance instrument.

Thermogravimetry (TG): A sample of LDPE-ester **3a** was subjected to a temperature ramp in the presence of a gas flow in a NETZSCH STA 449F3 STA449F3A-1625-M instrument. The thermal stability of LDPE-MA **3a** and pristine LDPE samples was determined using a TGA Auto Discovery 5500 Thermogravimetric Analyzer (TA Instruments). Briefly, approximately 8–15 mg of sample was heated from 20 to 900 °C at a ramp rate of 20 °C/min under a nitrogen atmosphere (50 mL/min).

Differential Scanning Calorimetry (DSC): Differential scanning calorimetry measurements were carried out using a Netzsch DCS300 Caliris calorimeter. Samples of about 10 mg weight sealed in aluminium pans where heated from 0 to 200 ºC at a scanning rate of 20 ºC/min under a nitrogen atmosphere to completely melt, eliminating the influence of thermal history, and then cooled to 40 ºC to obtain a crystallization process curve, and then heated to 200 ºC to obtain a melting curve. Then, the heating process for DSC runs twice.

Gas chromatography-mass spectrometry (GC-MS): Gas chromatographic analyses were performed in an instrument equipped with a 25 m capillary column of 5% phenylmethylsilicone. *N*-dodecane was used as an external standard. GC/MS analyses were performed on a spectrometer equipped with the same column as the GC and operated under the same conditions. Products were characterised by comparison with the given literature.

Gel Permeation Chromatography (GPC) Analysis of samples: The GPC experiments for LDPE samples were carried out using a GPC-IR Polymer Char-(IR5) equipment with 3 x PL gel Olexis Mix-Bed columns (13 microns), 300 x 7.5 mm with guard columns and an infrared detector (IR5). The IR5 is a high-resolution infrared detector to determine concentrations and composition in line, using continuous control of absorbance of chosen wave lengths. The experiments were conducted in a 1,2,4-trichlorobenzene (TCB) solution stabilized with antioxidant (300 ppm of butylhydroxytoluene (BHT)) at 160ºC, and at a flow rate of 1 mL/min. A single solution of each sample was prepared by adding 8 mL of eluent to 8 mg of original sample and heating 160ºC for 30-90 minutes as dissolution time. The solutions were filtered directly into autosampler vials, and injection of samples (200 µl) were carried out automatically. The esterified sample requires the filtration with EFS during 60 min and high stirring. Then, this sample solution was transferred through a disposable filter cartridge that removed the solid content. The result is a transparent solution collected in a 10 mL vial later analysed in the GPC-IR. Conventional results are based on peak position calibration of a series of narrow standards of known molar mass, obtained with an infrared detector. Results comply with the ISO 16014-4 and ASTM D6474 methods for high-temperature GPC measurement. To determine the number-average molar mass (M_n) and the molar mass distribution index (M_w/M_n) of the samples, the GPC system was

calibrated using a conventional calibration curve of third order made with 15 narrow polystyrene standards of know molecular weight (12900K g/mol to 672 g/mol) to determine molecular mass distribution and molecular averages, applying a Q factor to transform polystyrene values to PE values.

Contact angles and surface tension of LDPE samples: The surface tension and contact angles measurements were carried out by the *pendant* and the *sessile drop* methods, respectively, using distilled water and diiodomethane. The results were stablished on a Krüss DSA25 Expert contact-angle measuring goniometer and was found to agree well with literature values. Three measurements were made on each sample to obtain an average value for the entire surface, and standard deviation was found within 2º. The measurements were carried out using 60 µL of distilled water. The films were prepared by hot press molding at 110ºC and 150 kg/cm^3 pressure for 5 minutes.

Dynamic Mechanical Analysis of LDPE samples: Linear dynamic mechanical properties (DMA) measurements were carried out using a dynamic mechanical analyzer (DMA 2980 model equipment) from TA instruments on dual cantilever mode with rectangular specimens (dimensions: length 20 mm, width 10 mm and thickness around 5 mm). The temperature dependences of the storage flexural modulus E' and loss modulus E'', and loss tangent tan δ were measured from -100 to 200 ºC at a heating rate of 1 ºC/min. LDPE sample specimens are fabricated using a polymer injection molding (PIM) process machine (Ray Ran MPM-20 model). Operating and processing parameters of the PIM were optimized in a set of initial samples. The specimens were obtained with 10-15 grams of the desired LDPE sample using the ISO 1A halterion model, using the central part for the DMA measurements.

1.3 Reaction procedures and product characterization.

Reactions were generally performed either in 2.0 ml glass vials equipped with a magnetic stirrer and closed with a steel cap having a rubber septum part to sample out, or in 5-10 ml glass vials equipped with a magnetic stirrer and with an open reflux to recycle boiling solvents and allow volatiles to come out.

Yield calculations were double checked by the recovered weight and FT-IR employing calibration curves. For the latter, physical mixtures of a fixed amount of polyethylene with various relative weights of either lauryl ester or maleic anhydride were made and recorded, measuring the area of the characteristic peak in each case with respect to the sum of the area of the peaks at 728 cm^{-1} and 718 cm^{-1} of polyethylene, used as a normalization standard. The resulting relative areas were plotted against the percentage by weight of reactant in polyethylene and the described line was adjusted by least squares in order to obtain the corresponding equation (see Figures S2 and S18).

Synthesis of LDPE-3a and HDPE-3b: 250 mg of HDPE or LDPE and 0.5 mL of a 15% solution of ethyl diazoacetate (EDA, 5) in toluene (0.7 mmol) were placed in a 5 mL glass reactor, sealed and placed on a heating plate at 160 ºC for 24 h. Once the tube had been cooled, 1 mL of toluene was added to the mixture, which was magnetically stirred at 80 ºC for 1 h to dissolve the reaction mixture. On cooling, it was precipitated and filtered in vacuo, washing with diethyl ether. It was left under magnetic stirring with diethyl ether for 12 h to dissolve and remove by vacuum filtration the remains of ethyl diazoacetate. Finally, the solid was dried with a vacuum pump at room temperature for 12 h, to obtain 260 mg (97%) of **LDPE-3a** or 267 mg (>99%) of **HDPE-3b**. The product was characterized by FT-IR and NMR.

LDPE: FT-IR (υ, cm^{-1}): 2913, 2845, 1469, 1463, 728, 718.

LDPE-3a and **HDPE-3b:** FT-IR (υ, cm^{-1}): 2913, 2845, 1735, 1469, 1463, 728, 718. ^{1}H NMR (95 ºC, 400 MHz, toluene-d_8) δ: 4.06 (q, J = 6.8, Hz, 2H), 2.22 (d, J = 6.4 Hz, 2H), 1.97 (m, 1H), 1.36 (m, 303H), 1.10 (t, J = 6.8 Hz, 3H), 0.95 (m, 11H). ^{13}C NMR (100 MHz, toluene-d_8) δ: 171.4 (C), 60.0 (CH_2), 39.9 (CH_2), 38.4 (CH), 36.1 (CH), 35.0 (CH_2), 34.6 (CH_2), 31.0 (CH_2), 30.7 (CH_2), 30.5 (CH_2), 27.8 (CH_2), 27.3 (CH_2), 24.6 (CH_2), 23.7 (CH_2), 14.7 (CH_3), 14.6 (CH_3).

Synthesis of LDPE-carboxylate 4: 100 mg of LDPE-**3a** and 0.5 mL of a 12 M NaOH solution in 1.5 mL of toluene were introduced into a 5 mL glass reactor. The reactor is placed on a heating plate with magnetic stirring at 100 ºC for 48 h. On cooling, it was precipitated and washed several times with deionized water to remove residual base and filtered in vacuo washing with diethyl ether. Finally, it was dried with a vacuum pump at room temperature for 12 h, to obtain 98 mg (99%) of **LDPE-carboxylate 4**. The product was characterized by FT-IR.

LDPE-carboxylate 4 (υ, cm^{-1}): 2913, 2845, 1566, 1469, 1463, 728, 718.

Failed attempts of trans-esterification and trans-amidation of LDPE-3a: 50 mg of LDPE-**3a** were introduced into a 5 mL glass reactor, and then 100 mg of the corresponding alcohol, 0.5 mL of a 4 M or 12 M solution of hydrochloric acid or sodium hydroxide in 1.5 mL of toluene or 2-methyltetrahydrofuran, were added. The reactor is introduced into a heating plate with magnetic stirring at 100 ºC for 24 h. On cooling, it was precipitated and washed several times with deionized water to remove acid or base residues and the excess of reagents, and filtered under vacuum, washing with diethyl ether. Finally, it was dried with a vacuum pump at room temperature for 12 h. The resulting solids were characterized by FT-IR.

Esterification reaction of LDPE-carboxylate 4: 50 mg of LDPE-carboxylate **4**, 100 mg of the corresponding alcohol, and 0.5 mL of a 12 M hydrochloric acid solution in 1.5 mL of toluene, were introduced in a 5 mL glass reactor. The reactor was placed in a heating plate with magnetic stirring at 100 ºC for 24 h. On cooling, it was precipitated with deionized water and washed several times, to remove the residual acid and excess reagent, and filtered under vacuum, washing with diethyl ether. Finally, it was dried with a vacuum pump at room temperature for 12 h, to obtain typically 50 mg (>98%). The products were characterized by FT-IR.

LDPE-oleate 5 and **LDPE-2-ethylhexanoate 6** (same signals; υ, cm^{-1}): 2913, 2845, 1735, 1469, 1463, 728, 718.

Amidation reaction of LDPE-carboxylate 4: 50 mg of LDPE-carboxylate **4** were added to a 5 mL glass reactor with 100 mg of the corresponding amine in 1.5 mL of toluene, and 0.5 mL of a 4 M or 12 M hydrochloric acid solution were added. The reactor was placed in a heating plate with magnetic stirring at 100 ºC for 24 h. On cooling, it was precipitated with deionized water and washed several times to remove residual acid, base and excess reagents, and filtered under vacuum, washing with diethyl ether. Finally, it was dried with a vacuum pump at room temperature for 12 h, to obtain 41 mg (80%) of **7** and 48 mg (95%) of **8**. The resulting solids were characterized by FT-IR.

LDPE-oleylamide 7 and **LDPE-2-ethylhexylamide 8** (same signals; υ, cm^{-1}): 2913, 2845, 1609, 1485, 1469, 1463, 728, 718.

Hydrolysis tests for functionalized LDPE with EDA: 10 mg of functionalized LDPE were introduced in a 1 mL glass reactor, and 0.5 mL of a 1 M solution of HCl (pH = 1) or NaOH (pH = 14) were added at room temperature. The mixture was magnetically stirred at RT or 100 ºC for 24 h. After this time, the solid was washed several times with deionized water to remove traces of acid or base, and filtered under vacuum washing with diethyl ether. Finally, it was dried with a vacuum pump at room temperature for 12 h, to obtain typically 10 mg of solid. The resulting solids were characterized by FT-IR.

Synthesis of LDPE-13a and HPDE-13b without solvent: In a 5 mL glass vial, 250 mg of either high (HDPE) or low (LDPE) density polyethylene, 25 mg (10% by weight) of maleic anhydride (MA) and 5 mg (2% by weight) of dicumyl hydroperoxide (DCP, as a radical initiator) were introduced. The reactor was placed on a hot plate at 160 ºC for 24 h. Once at room temperature, 1 mL of toluene was added and left under magnetic stirring at 80 °C for 1 h to dissolve the reaction mixture. On cooling, it was precipitated and filtered in vacuo, washing with water. It was left under magnetic stirring with water at 50 ºC for 1 h. Finally, it was filtered under vacuum, washing with acetone and drying with a

vacuum pump at room temperature for 12 h, to obtain 251 mg of **LDPE-13a** or 254 mg of **HDPE-13b**. The resulting solids was characterized by FT-IR and NMR.

LDPE-MA 13a and **HDPE-MA 13b** (same signals, υ, cm^{-1}): 2913, 2845, 1780, 1735, 1706, 1469, 1463, 728, 718.

Synthesis of LDPE-13a and HPDE-13b with solvent: The same procedure than above was followed, but adding 1 mL of toluene or mesitylene as a solvent. The reactor was placed on a hot plate at 100 or 130 ºC for 24 h. On cooling, the same work-up than above was employed, to obtain 254 mg (92%) of **LDPE-13a** or 269 mg (98%) of **HDPE-13b**. The product was characterized by FT-IR and SEM.

Synthesis of 100 grams of LDPE-MA 13a: In a 500 mL glass vial, 100 g of low density polyethylene (LDPE), 10 g (10% by weight) of maleic anhydride (MA) and 2 g (2% by weight) of dicumyl hydroperoxide (DCP, as a radical initiator) and 300 mL of mesitylene as a solvent were introduced. The reactor was placed on a hot plate at 130 ºC for 24 h. On cooling, it was precipitated and filtered in vacuo, washing with water. It was left under magnetic stirring with water at 50 ºC for 1 h. Finally, it was filtered under vacuum, washing with acetone and drying with a vacuum pump at room temperature for 12 h.

Synthesis of re-precipitated LDPE: In a 500 mL glass vial, 100 g of low density polyethylene (LDPE) and 300 mL of mesitylene as a solvent were introduced. The reactor was placed on a hot plate at 130 ºC for 24 h. On cooling, it was precipitated and filtered in vacuo, washing with water. It was left under magnetic stirring with water at 50 ºC for 1 h. Finally, it was filtered under vacuum, washing with acetone and drying with a vacuum pump at room temperature for 12 h.

Synthesis of LDPE-15: 100 mg of maleic anhydride-functionalized low-density polyethylene (LDPE-MA **13a**) was introduced into a 5 mL glass reactor and 2 mL of a 6 M solution of NaOH in 4 mL of toluene was added, and the mixture was magnetically stirred at 100 °C for 24 h. After this time, the solid was precipitated with deionized water and washed several times, to remove base residues, vacuum filtered by washing with acetone, and dried with a vacuum pump at room temperature for 12 h, to obtain 97 mg (98%) of **LDPE-15**. The product was characterized by FT-IR.

LDPE-15 (υ, cm^{-1}): 2913, 2845, 1706, 1566, 1469, 1463, 728, 718.

Esterification reaction of LDPE-MA 13a: 50 mg of LDPE-MA **13a**, 100 mg of the corresponding alcohol, and 0.5 mL of a 12 M hydrochloric acid or sodium hydroxide solution in 1.5 mL, were introduced into a 5 mL glass reactor with toluene. The reactor was placed in a heating plate

under magnetic stirring at 100 ºC for 24 h. On cooling, it was precipitated with deionized water and washed several times to remove residual acid, base and excess reagent, and filtered under vacuum, washing with diethyl ether. Finally, it was dried under vacuum at room temperature for 12 h, to obtain typically 45 mg (~80%) of solid. The products were characterized by FT-IR.

LDPE-oleate 16 and **LDPE-2-ethylhexanoate 17** (same signals; υ, cm^{-1}): 2913, 2845, 1735, 1706, 1469, 1463, 728, 718.

Amidation reaction of LDPE-MA 13a: 50 mg of LDPE- MA **13a**, 100 mg of the corresponding amine in 1.5 mL of toluene, and 0.5 mL of a 4-12M hydrochloric acid solution, or 0.5 mL of a 6M sodium hydroxide solution, were placed in a 5 mL glass reactor. The reactor was then placed in a heating plate under magnetic stirring at 100 ºC for 24 h. On cooling, it was precipitated and washed several times with deionized water, in order to remove the residual acid, base and excess reagents, and the mixture was filtered under vacuum, washing with diethyl ether. Finally, the resulting solid was dried with a vacuum pump at room temperature for 12 h, to obtain typically 43 mg (~75%) of solid. The products were characterized by FT-IR.

LDPE-oleylamide 18 and **LDPE-2-ethylhexilamide 19** (same signals; υ, cm^{-1}): 2913, 2845, 1609, 1485, 1469, 1463, 728, 718. (υ, cm^{-1}): 2913, 2845, 1609, 1485, 1469, 1463, 728, 718.

Synthesis of LDPE-acrylates 20 and 21: 100 mg of LDPE, 25 mg (10% by weight) of the corresponding acrylate and 5 mg (2% by weight) of DCP were introduced into a 5 mL glass reactor. The reaction mixture was placed on a hot plate at 160 ºC for 24 h. Once the flask was cooled, 1 mL of toluene was added and left under magnetic stirring at 80 ºC for 1 h to dissolve the reaction mixture. On cooling, it was precipitated and filtered in vacuo washing with diethyl ether. Finally, it was dried with a vacuum pump at room temperature for 12 h. The resulting solids were characterized by FT-IR.

LDPE-2-ethylhexanoate 20 and **LDPE-benzoate 21** (same signals; υ, cm^{-1}): 2913, 2845, 1735, 1706, 1469, 1463, 728, 718. (υ, cm^{-1}): 2913, 2845, 1735, 1706, 1469, 1463, 728, 718.

2 Tables.

Table S1 Fourier transformed infrared (FTIR) signals for the ester-functionalized **LDPE-3a**, with assignations.

**Signal (cm^{-1})**	**Assignation**
2900	C-H stretching
1856	C=O asymmetric stretching, maleic acid (MA)
1780	C=O symmetric stretching, maleic acid (MA)
1735	C=O symmetric stretching, ester
1706	C=O symmetric stretching, carboxylic acid
1609	C=O bending, amide
1566	C=O bending, carboxylate
1485	C=O bending, amide

Table S2 Results for the elemental analysis of the LDPE-ester **3a** polymer after washings with diferent solvents under agitation at room temperature for 18 h.

**Washing solvent**	**Remaining N (%)**
Et_2O	0.061
DCM	0.056
ACN	0.081
H_2O	0.315

3 Figures.

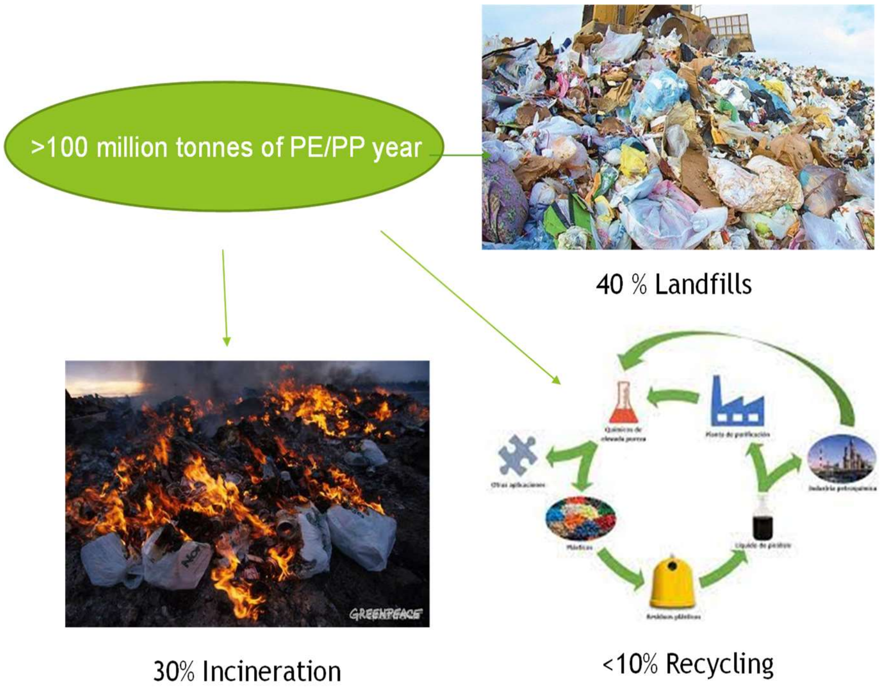

Figure S1 Current polyethylene (LDPE and HDPE) and polypropylene (PP) disposal.

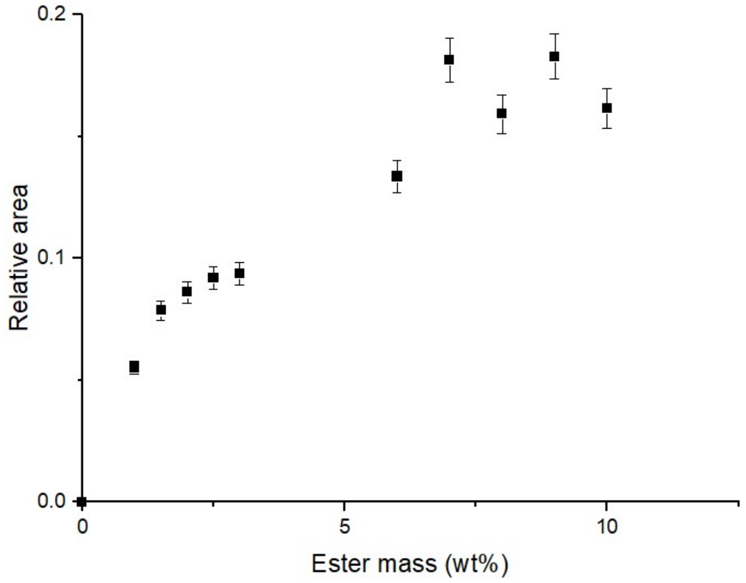

Figure S2 Fourier transformed-infrared (FT-IR) calibration for PE-ester **3a**. Error bars account for a 5% uncertainty.

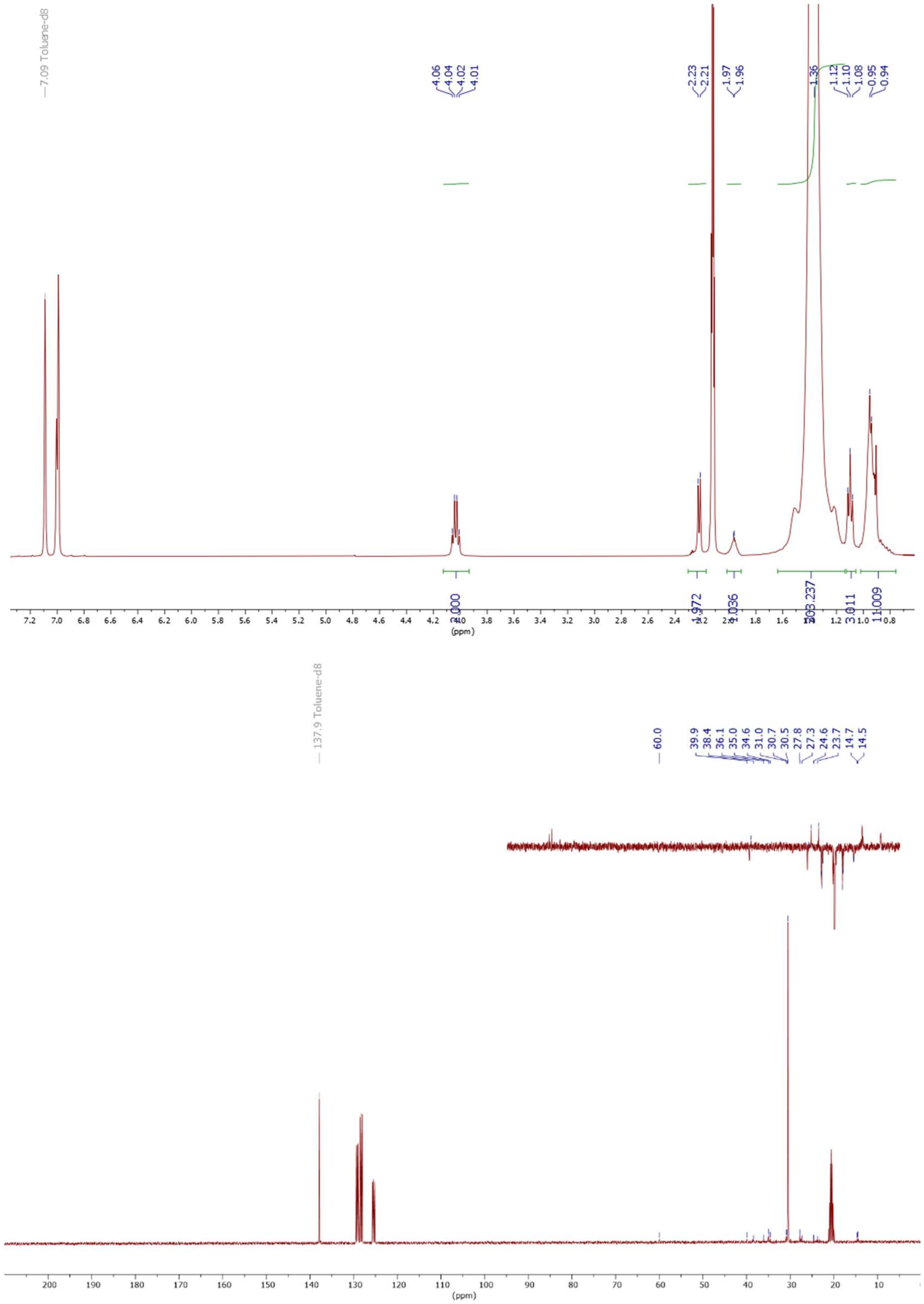

Figure S3 ^{1}H (a) and ^{13}C (b) NMR spectra of LDPE-ester **3a** in deuterated toluene at 70-95 ºC. The inset shows the corresponding DEPT spectrum.

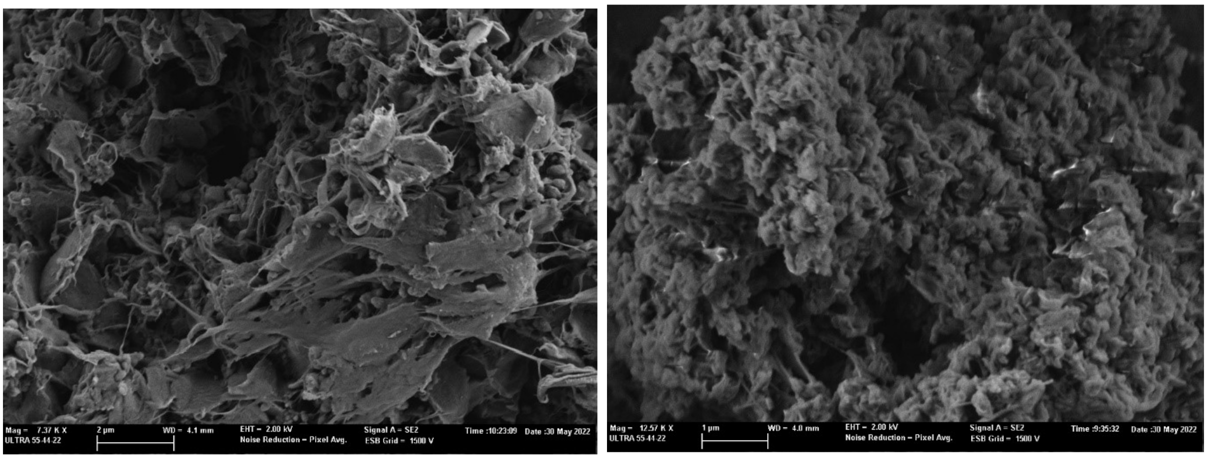

Figure S4 Scanning electron microscopy (SEM) images of LDPE **1a** (left) and LDPE-ester **3a** (right).

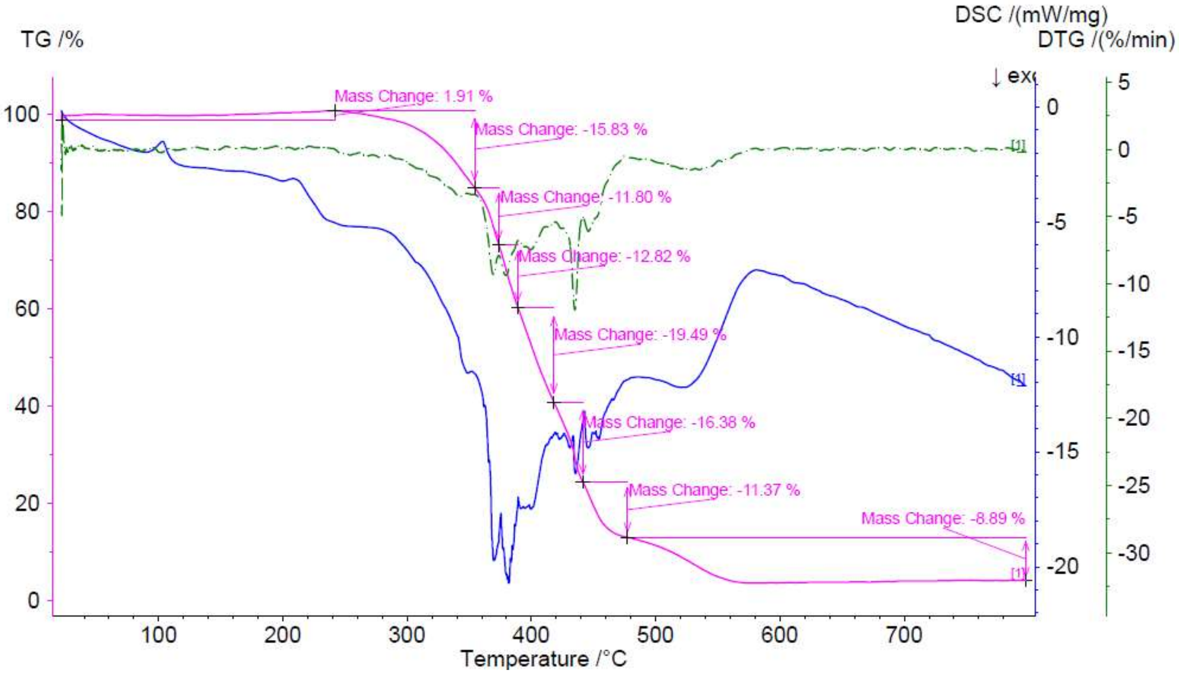

Figure S5 Thermogravimetric analysis (TGA), differential scanning calorimetry (DSC) analysis and DTG of LDPE-ester **3a**.

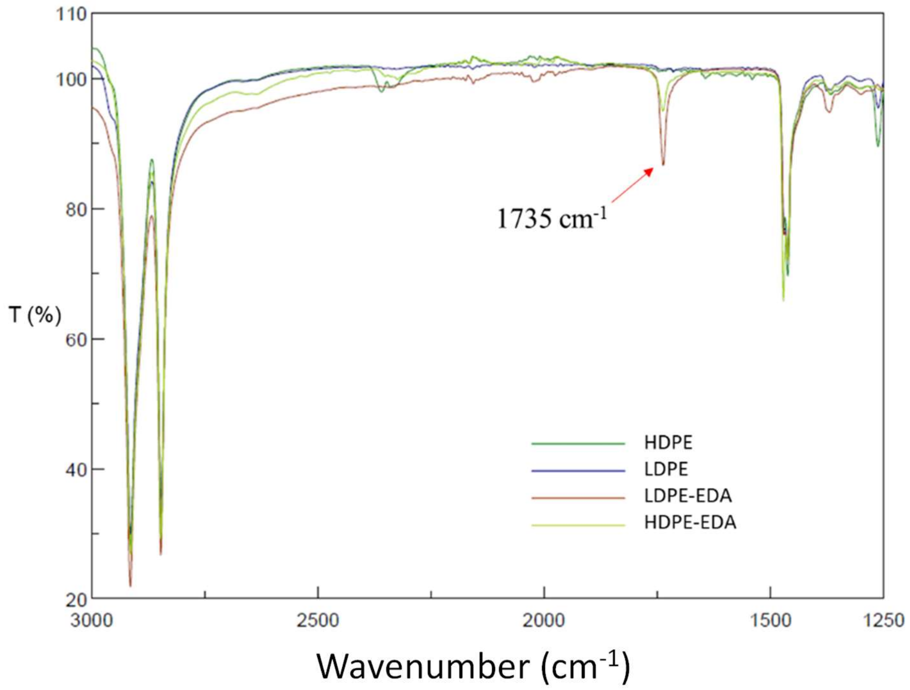

Figure S6 Fourier transformed-infrared (FT-IR) spectra of LDPE, HDPE, and the corresponding PE-esters **3a** and **3b**.

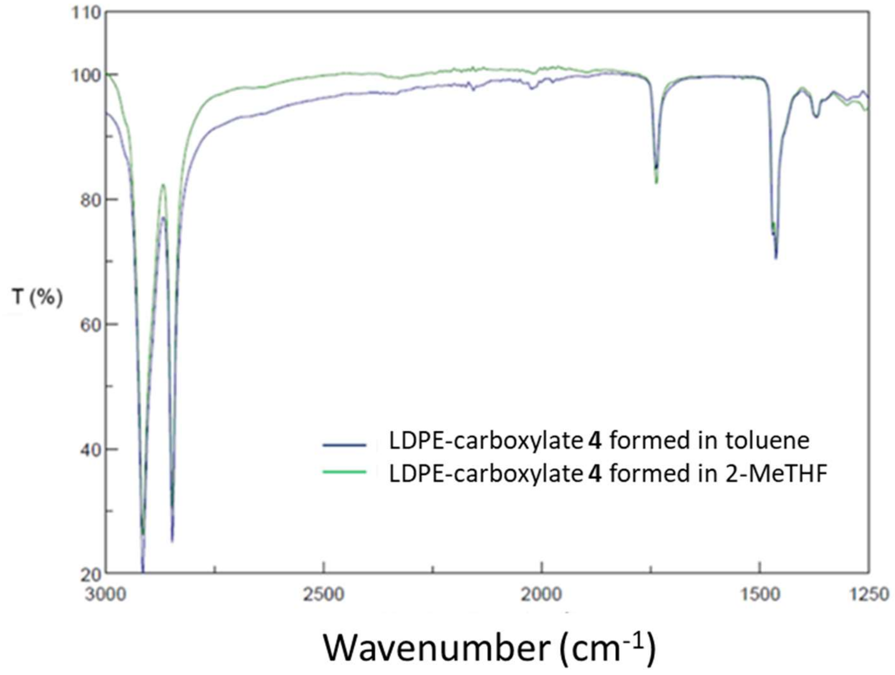

Figure S7 Fourier transformed-infrared (FT-IR) spectra of LDPE-carboxylate **4** formed either in toluene or 2-methyltetrahydrofurane solvent under similar reaction conditions.

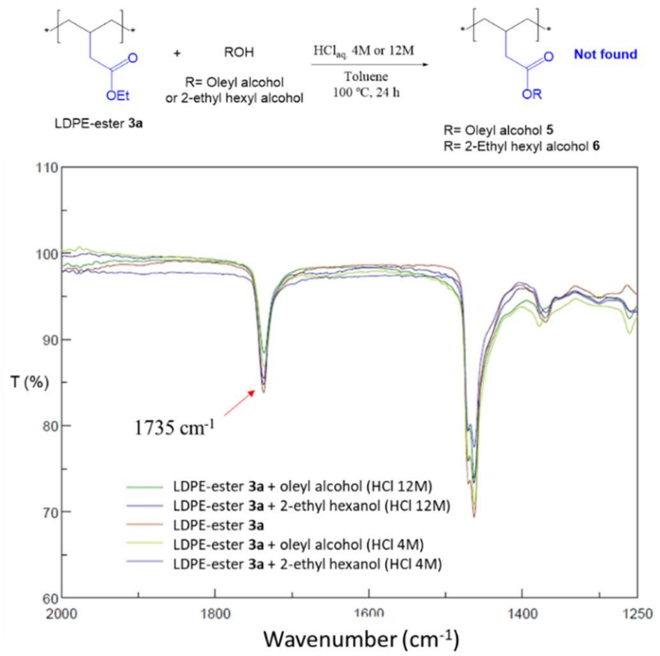

Figure S8 Fourier transformed-infrared (FT-IR) spectra of LDPE-ester **3a** under different acid-catalyzed transesterification reaction conditions with oleyl alcohol and 2-ethyl hexyl alcohol.

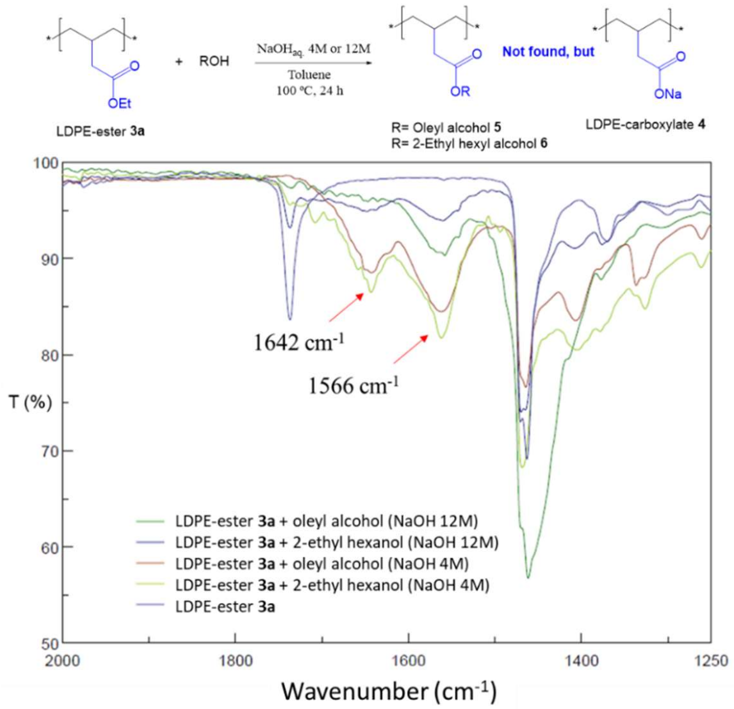

Figure S9 Fourier transformed-infrared (FT-IR) spectra of LDPE-ester **3a** under different base-catalyzed transesterification reaction conditions with oleyl alcohol and 2-ethyl hexyl alcohol. The peak at 1566 cm^{-1} is indicative of the carboxylate and the new peak at 1642 cm^{-1} corresponds to hydrogen bridging in the aqueous media (sample not dried).

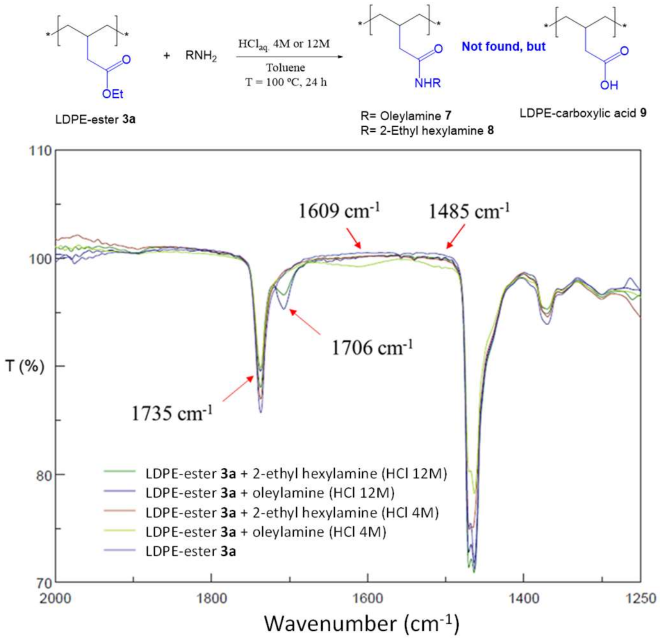

Figure S10 Fourier transformed-infrared (FT-IR) spectra of LDPE-ester **3a** under different acid-catalyzed transesterification reaction conditions with oleylamine and 2-ethyl hexylamine. The peaks at 1609 cm^{-1} and 1485 cm^{-1} correspond to amides, and the peak at 1706 cm^{-1} corresponds to the carboxylic acid.

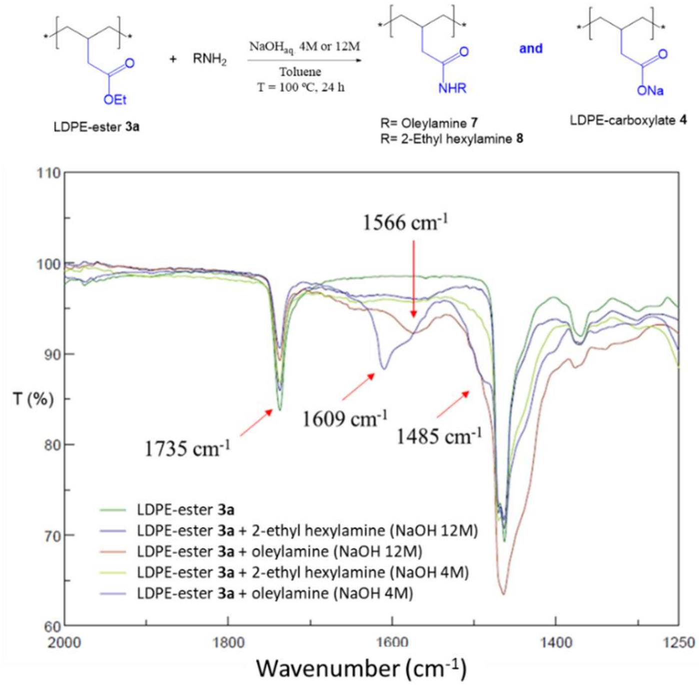

Figure S11 Fourier transformed-infrared (FT-IR) spectra of LDPE-ester **3a** under different base-catalyzed transesterification reaction conditions with oleylamine and 2-ethyl hexylamine. The peaks at 1609 cm^{-1} and 1485 cm^{-1} correspond to amides, and the peak at 1566 cm^{-1} corresponds to the carboxylate.

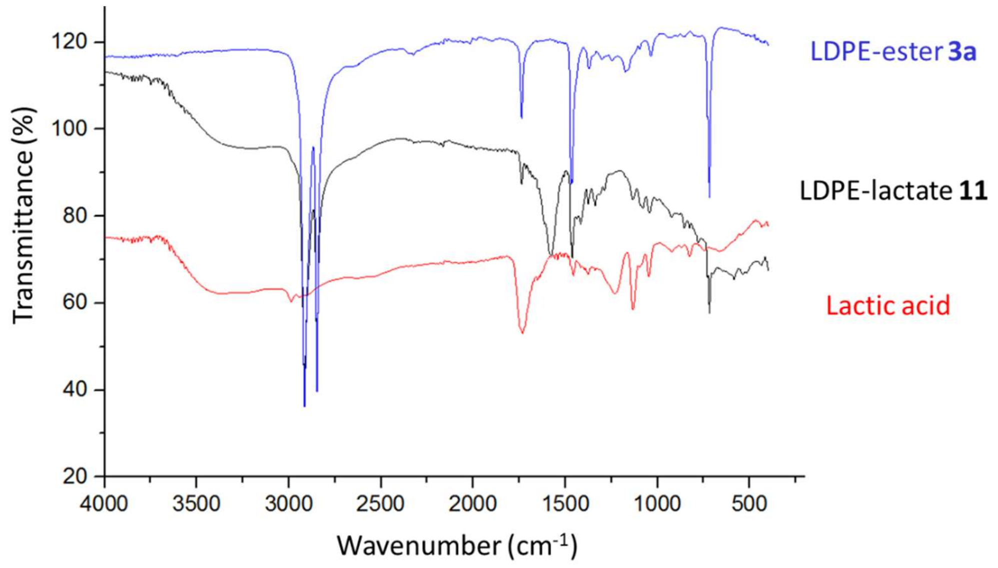

Figure S12 Fourier transformed infrared (FTIR) spectra of LDPE-ester **11** after extensive washing of the solid material with toluene, water and acetone, and LDPE-ester **3a** and lactic acid.

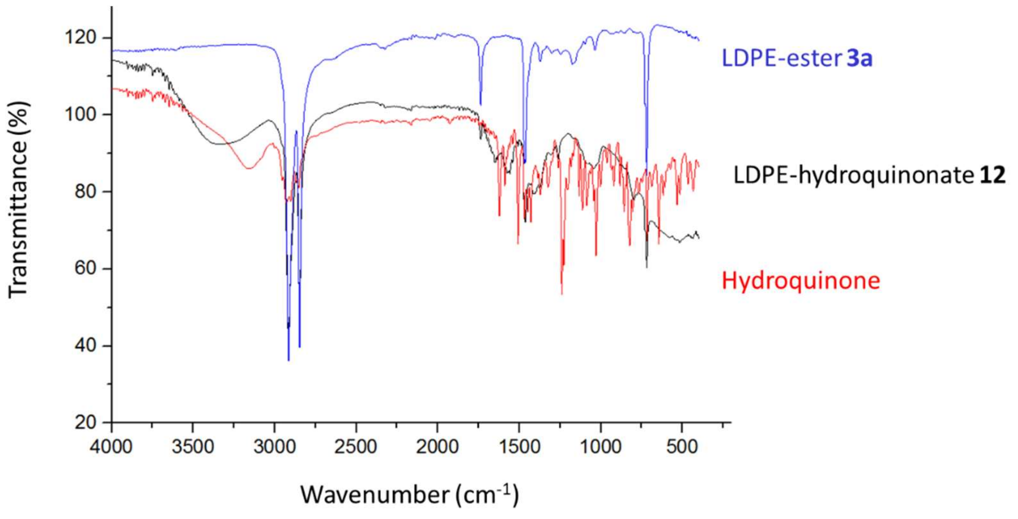

Figure S13 Fourier transformed infrared (FTIR) spectra of LDPE-ester **12** after extensive washing of the solid material with toluene, water and acetone, and LDPE-ester **3a** and hydroquinone.

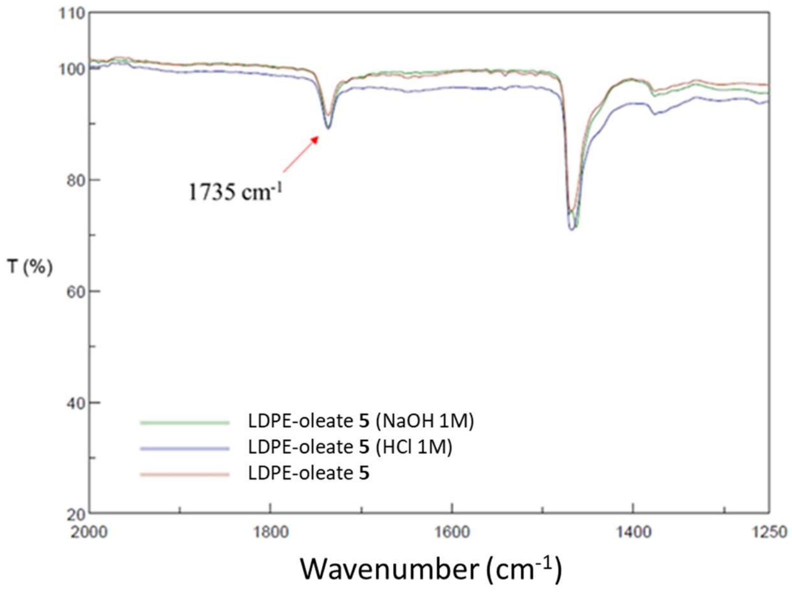

Figure S14 Fourier transformed infrared (FTIR) spectra of LDPE-ester **5** after treatment with 1M NaOH or 1M HCl in boiling water (100 ºC) for 1 h.

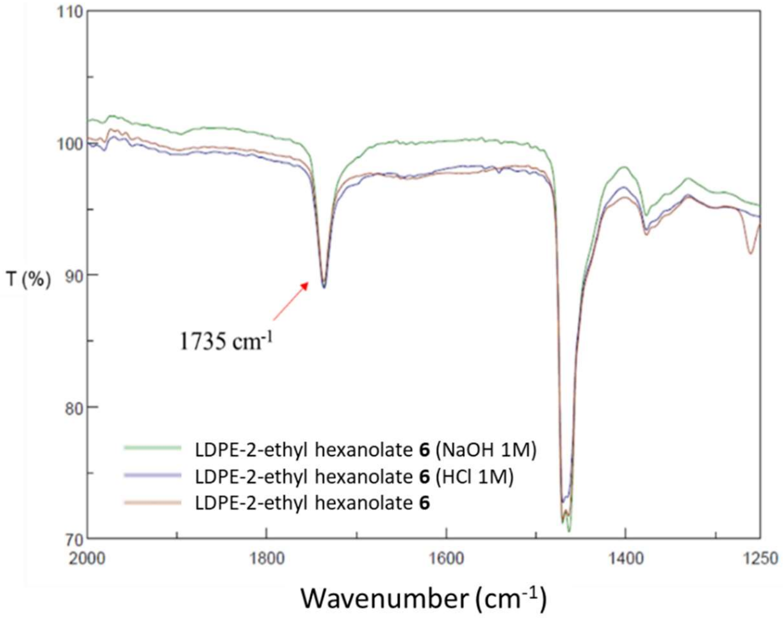

Figure S15 Fourier transformed infrared (FTIR) spectra of LDPE-ester **6** after treatment with 1M NaOH or 1M HCl in boiling water (100 ºC) for 1 h.

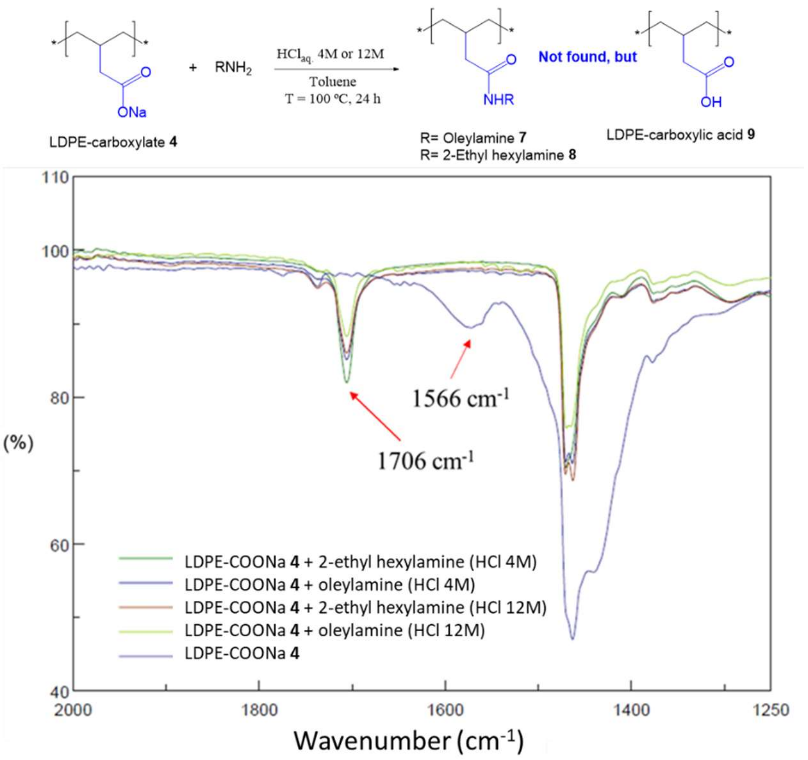

Figure S16 Fourier transformed infrared (FTIR) spectra of LDPE-carboxylate **4** (LDPE-COONa) after transamidation reaction with different amines under acid conditions. The peak at 1706 cm^{-1} corresponds to the carboxylic acid.

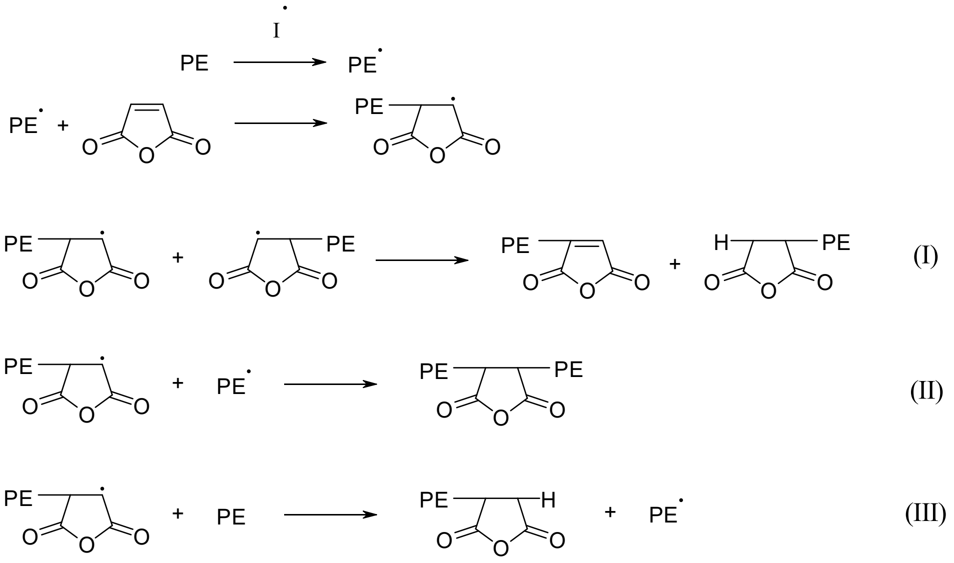

Figure S17 Described radical mechanism for the incorporation of MA in PE (ref. 5 in the text).

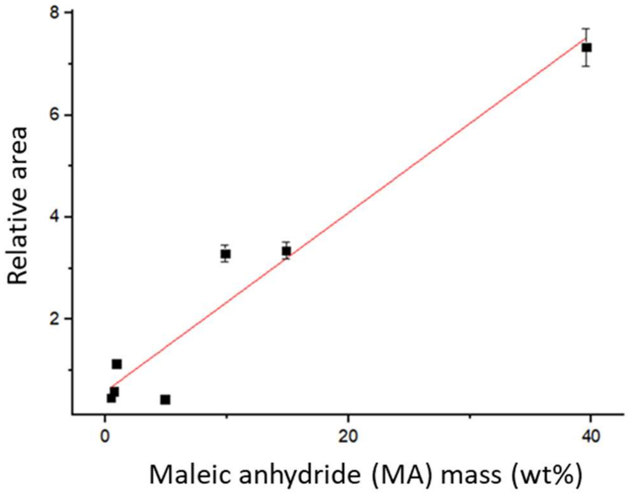

Figure S18 Fourier transformed-infrared (FT-IR) calibration of PE-MA **13a**. Error bars account for a 5% uncertainty.

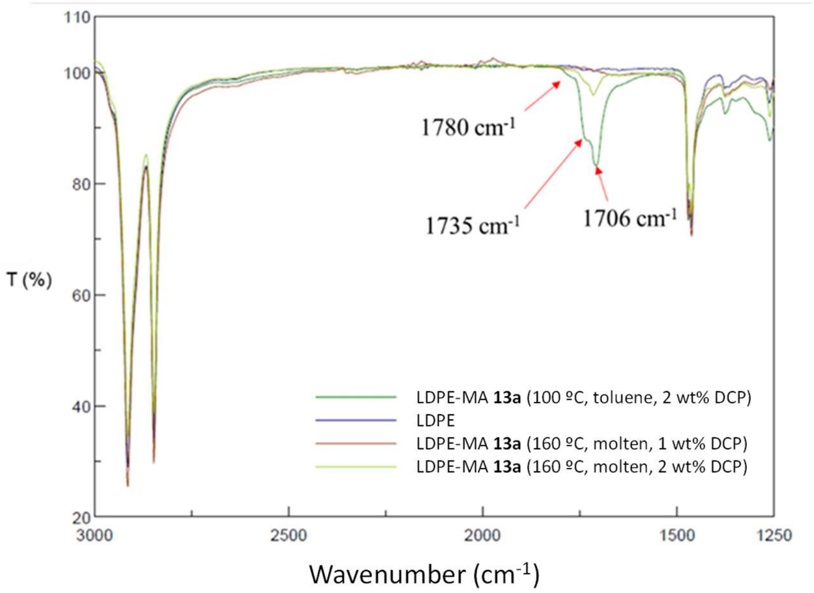

Figure S19 Fourier transformed infrared (FTIR) spectra of LDPE reacted with MA under different radical reaction conditions. The peaks at 1780 cm^{-1} and 1735 cm^{-1} correspond to vibrations of MA and the peak at 1706 cm^{1} corresponds to hydrolyzed MA (carboxylic acid).

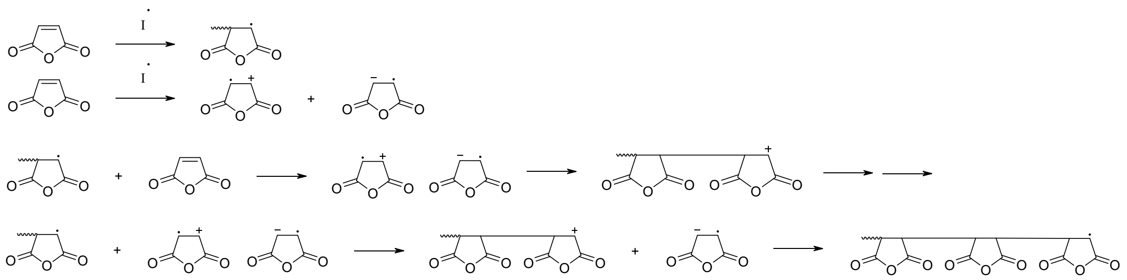

Figure S20 Radical homopolymerization of MA.

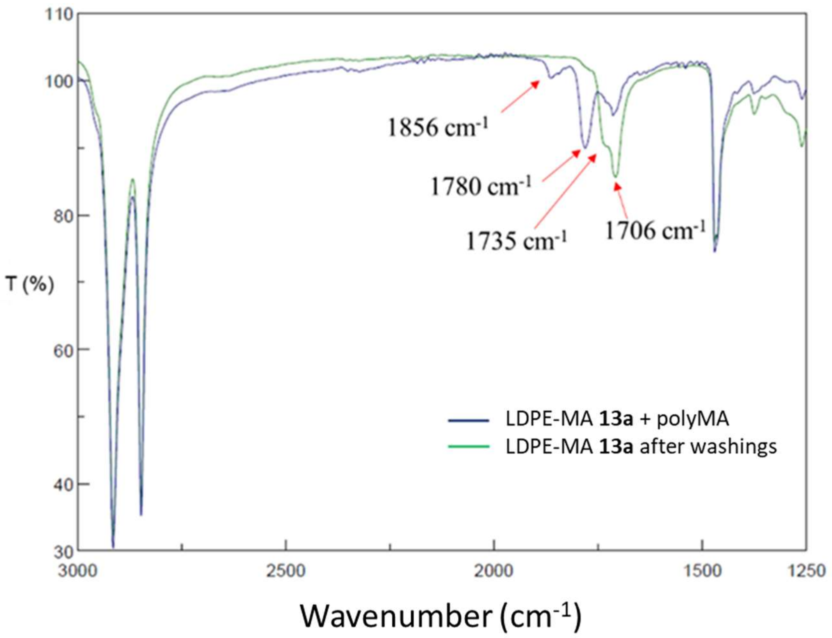

Figure S21 Fourier transformed infrared (FTIR) spectra of LDPE-MA **13a** before and after washing with boiling water. The peaks at 1856 cm^{-1} and 1780 cm^{-1} are associated to asymmetric and symmetric stretching vibrations, respectively, of the anhydride group in polyMA **14**.

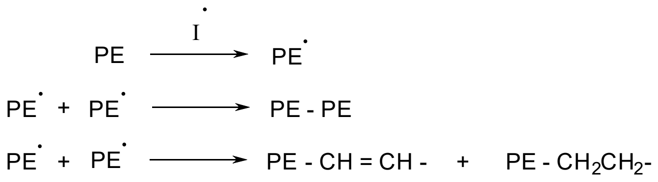

Figure S22 Radical intercrossing of PE during functionalization with MA. This process must be more severe for LDPE than for HDPE.

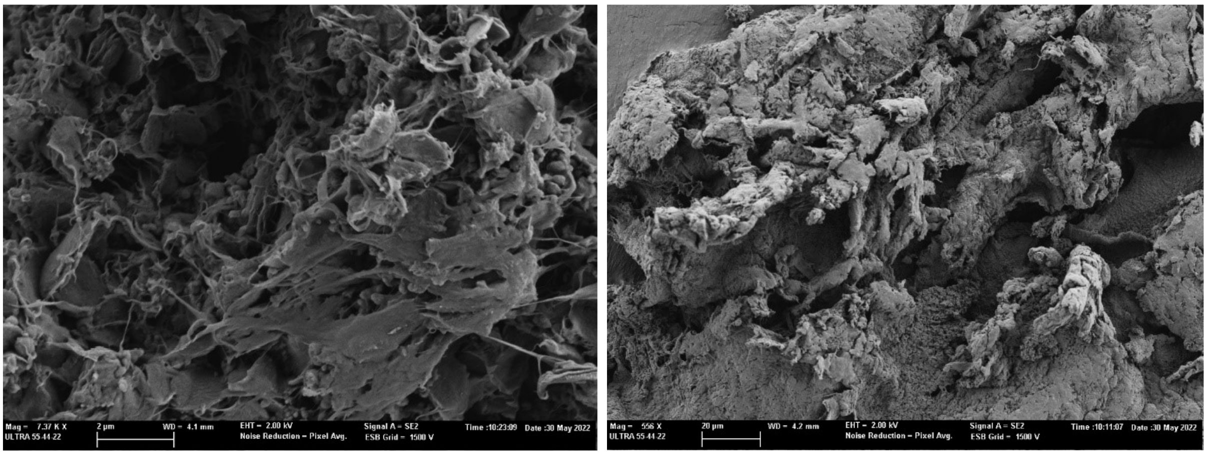

Figure S23 Scanning electron microscopy (SEM) images of LDPE (left) and LDPE-MA **13a** (right), showing the higher density of the new material.

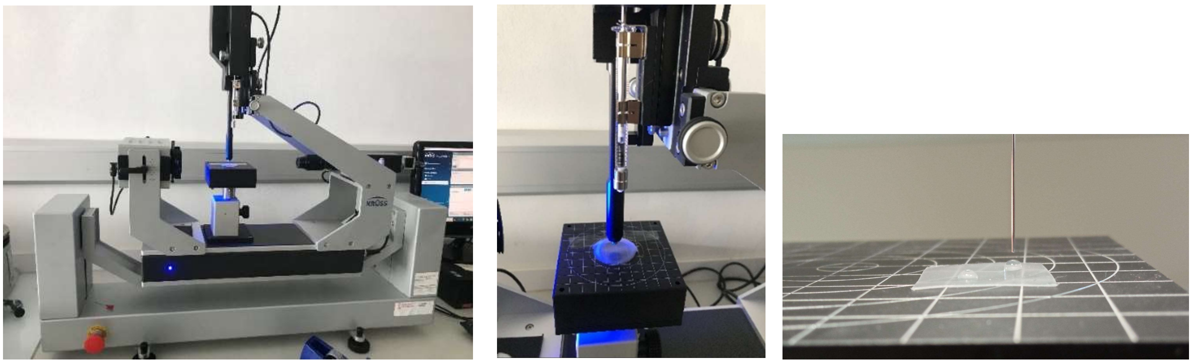

Figure S24 Krüss DSA25 expert contact-angle measuring system employed in this work and sample treatment.

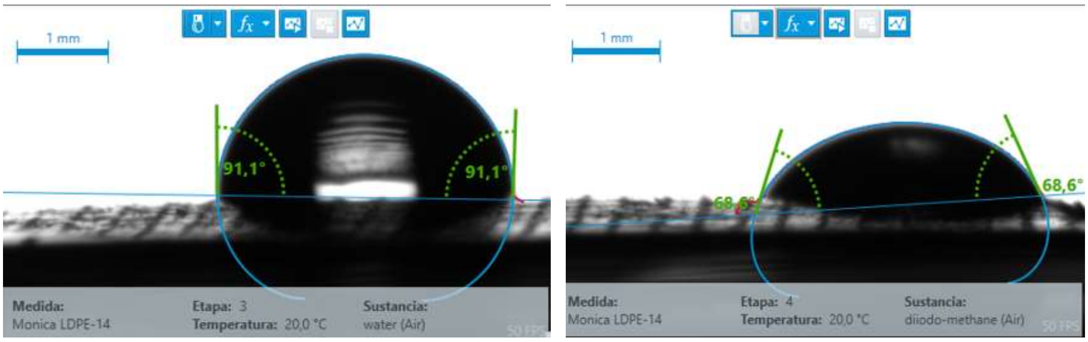

Figure S25 Measurement of a LDPE precipitated sample at 20 °C using water (a) or diiodomethane (b) liquids.

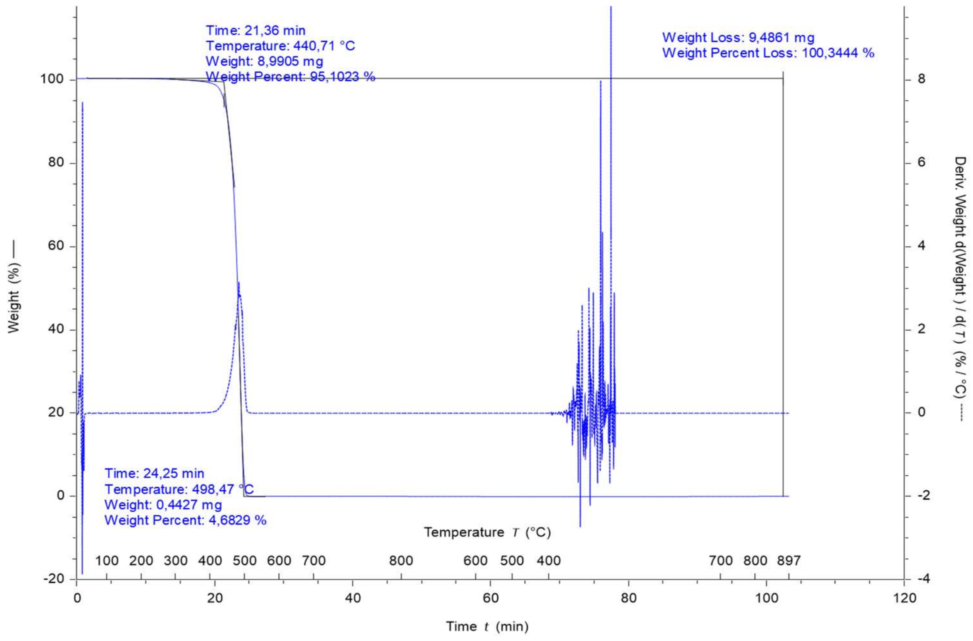

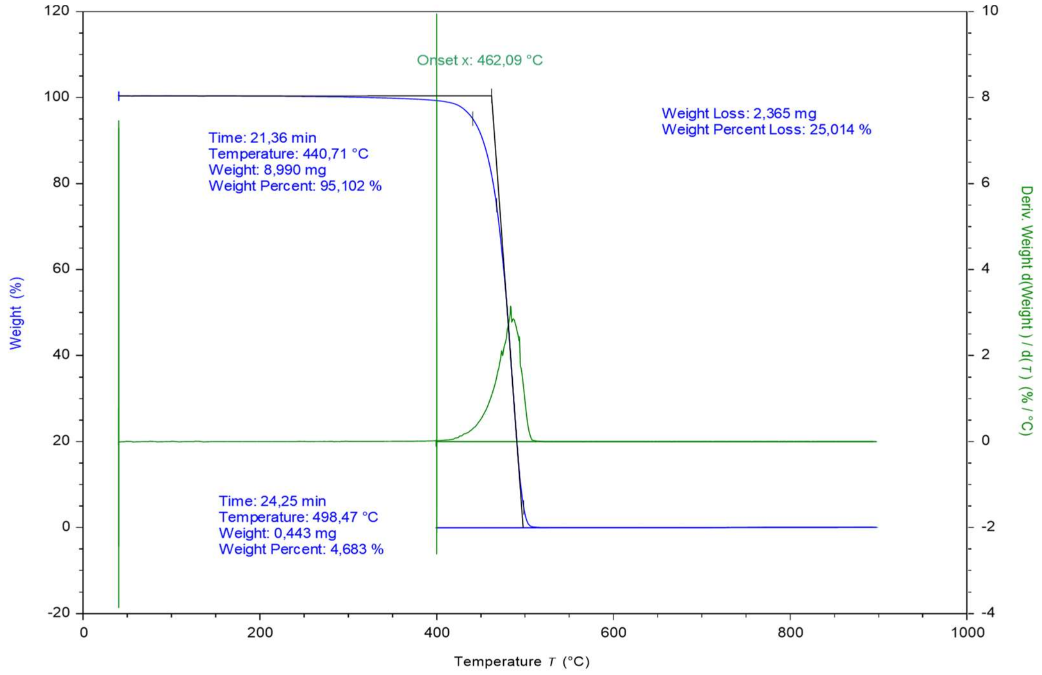

Figure S26 Thermogravimetric analysis (TGA) of pristine LDPE.

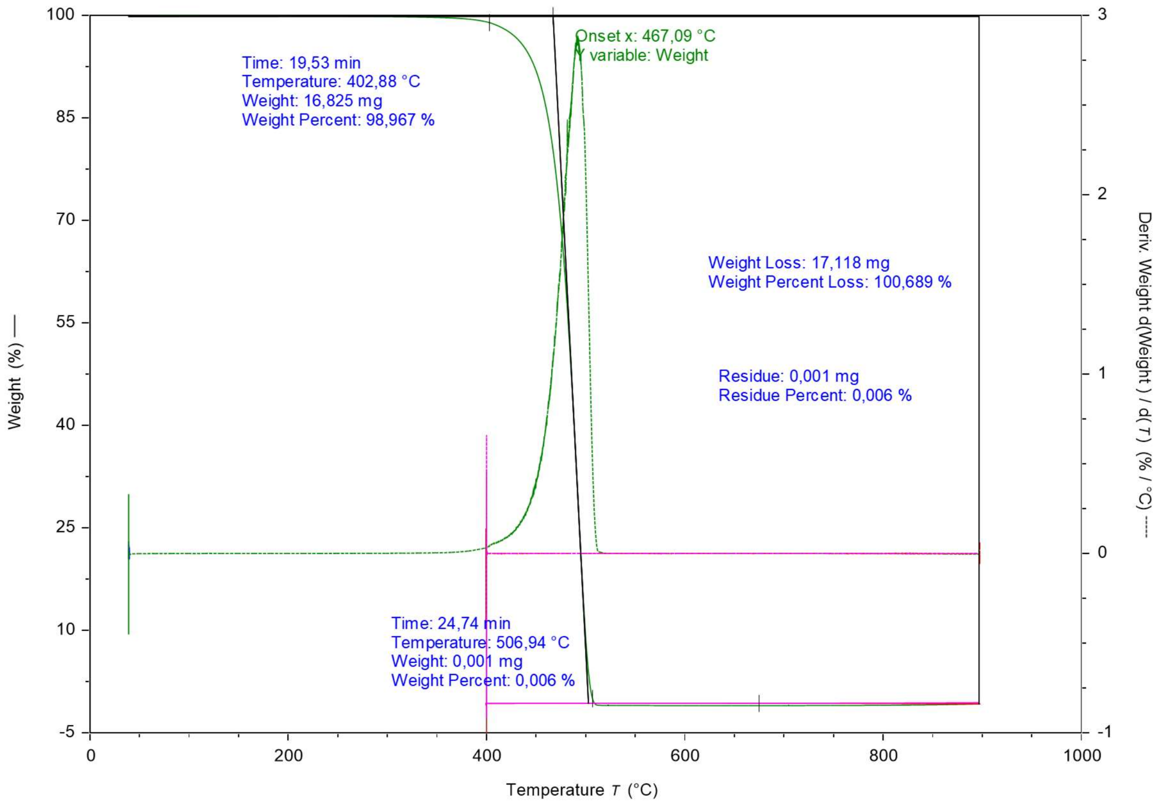

Figure S27 Thermogravimetric analysis (TGA) of precipitated LDPE.

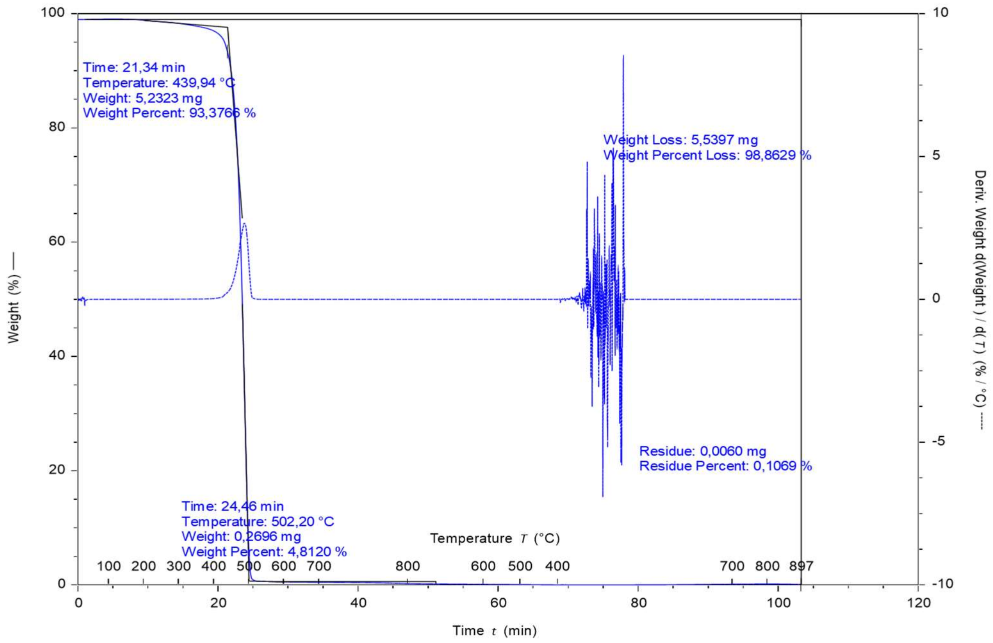

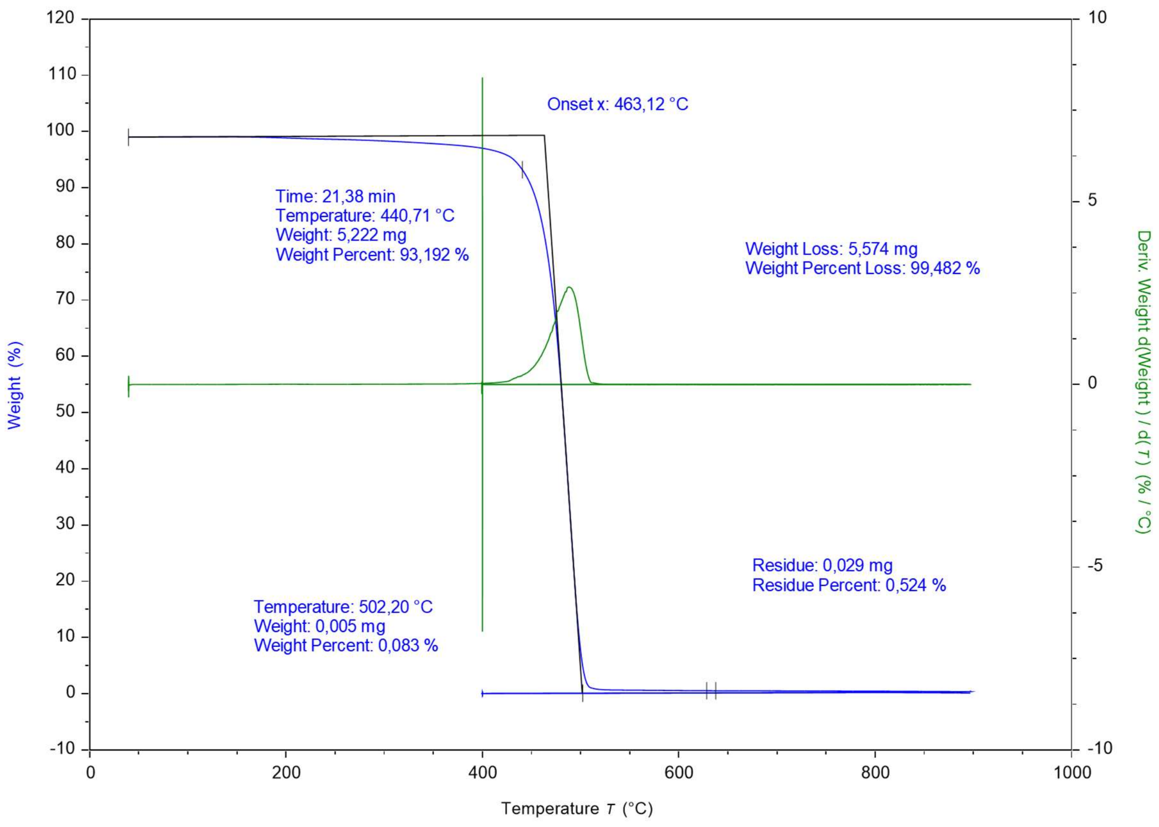

Figure S28 Thermogravimetric analysis (TGA) of LDPE-MA **13a**.

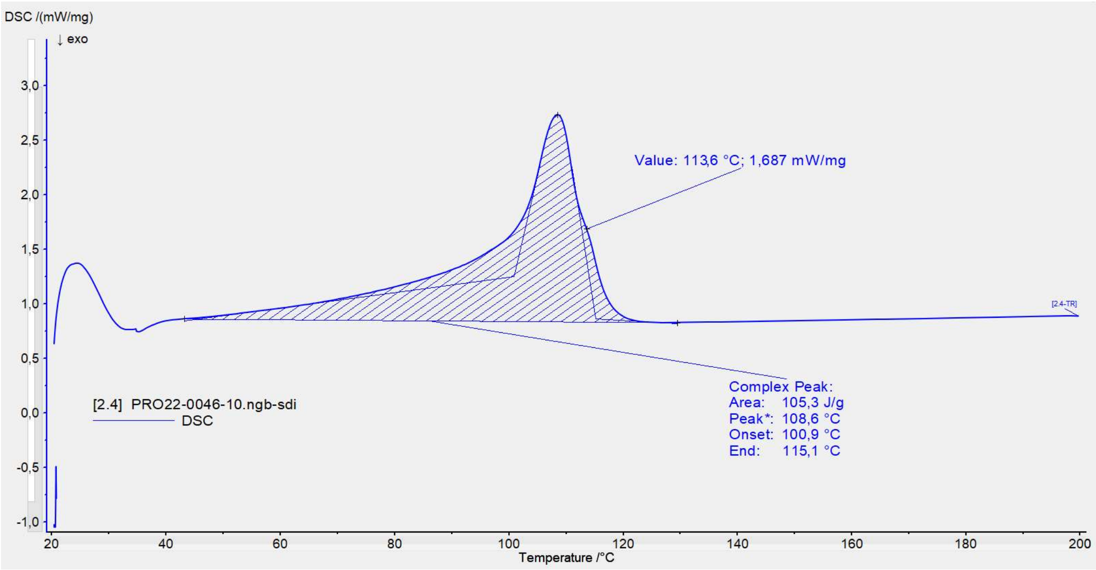

Figure S29 Differential scanning calorimetry (DSC) of LDPE-MA **13a** (second heating)

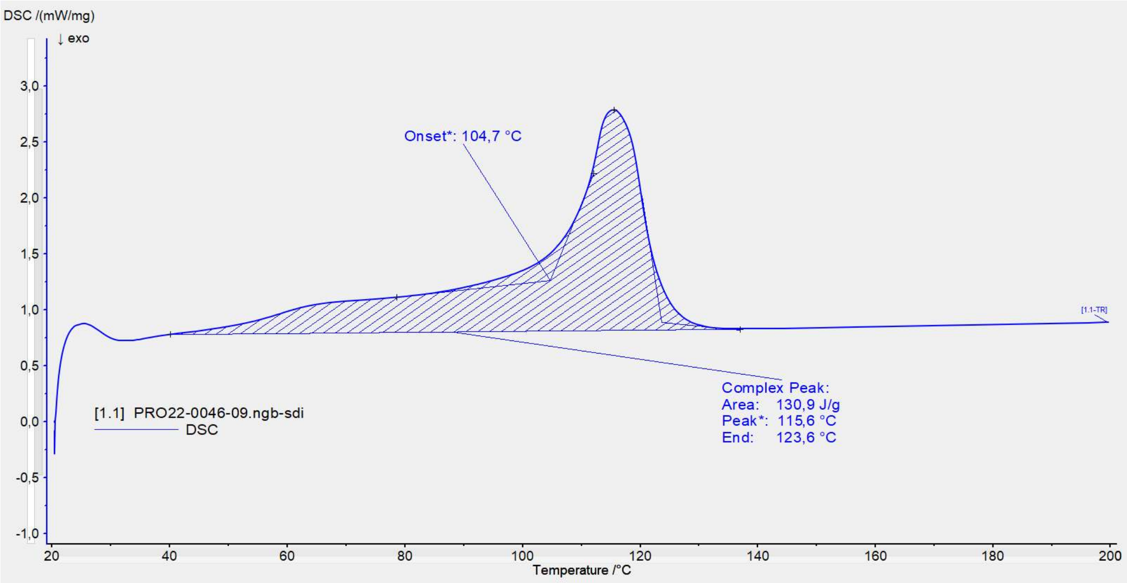

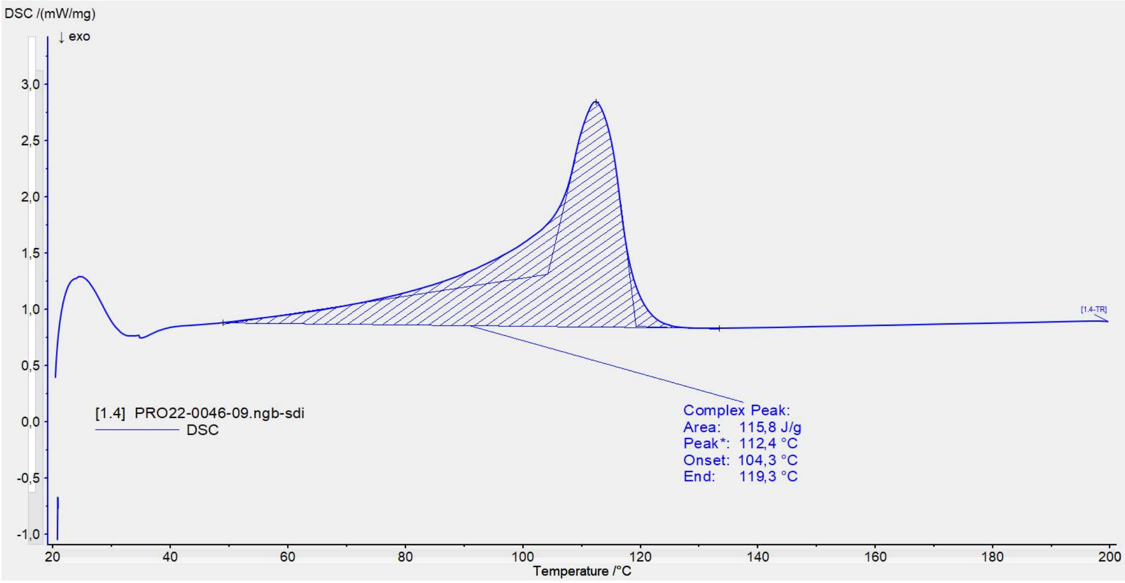

Figure S30 Differential scanning calorimetry (DSC) of pristine LDPE, first (top) and second heating (bottom)

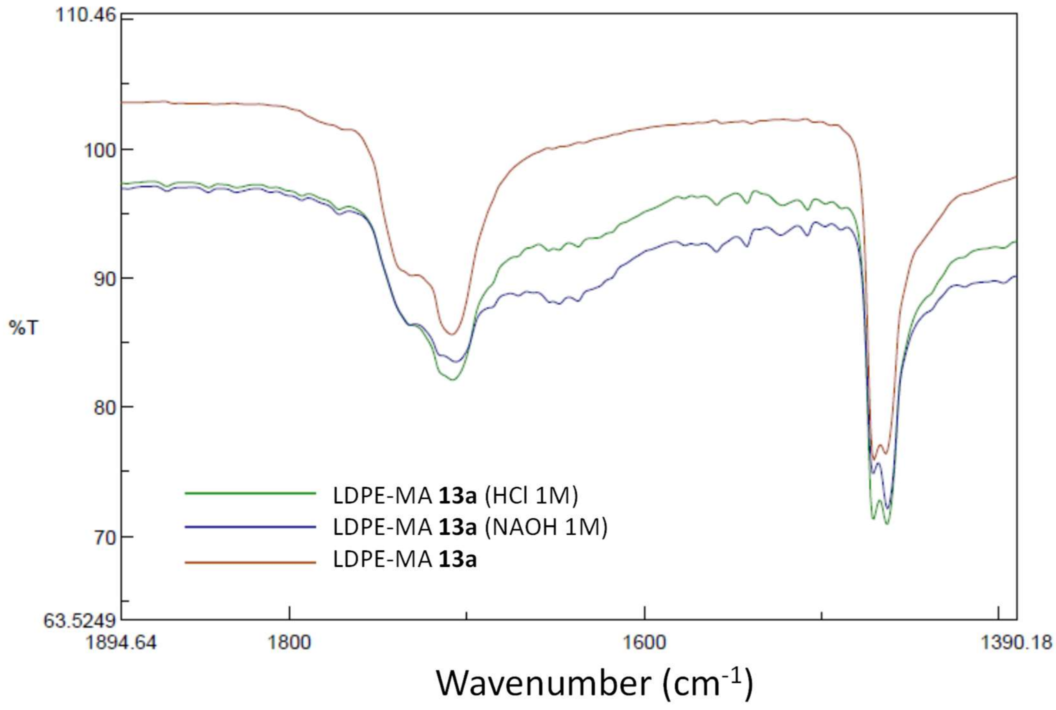

Figure S31 Fourier transformed infrared (FTIR) spectra of LDPE-MA **13a** after treatment with 1M HCl or 1M NaOH at 25 ºC for 24 h. No signs of degradation are detected.

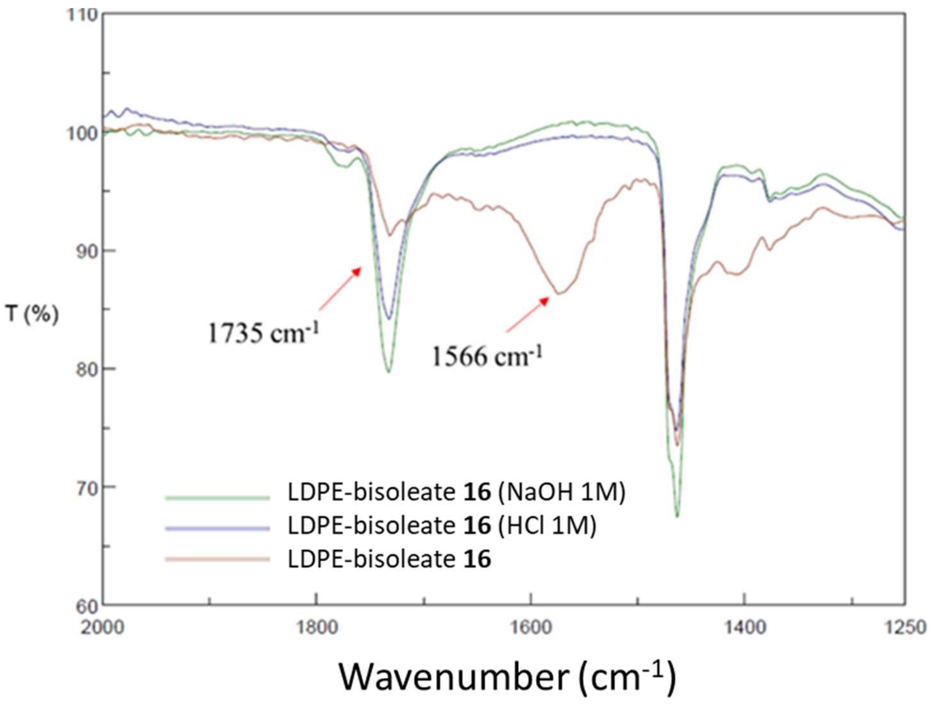

Figure S32 Fourier transformed infrared (FTIR) spectra of LDPE-bisoleate **16** after treatment with 1M NaOH or 1M HCl in boiling water (100 ºC) for 1 h.

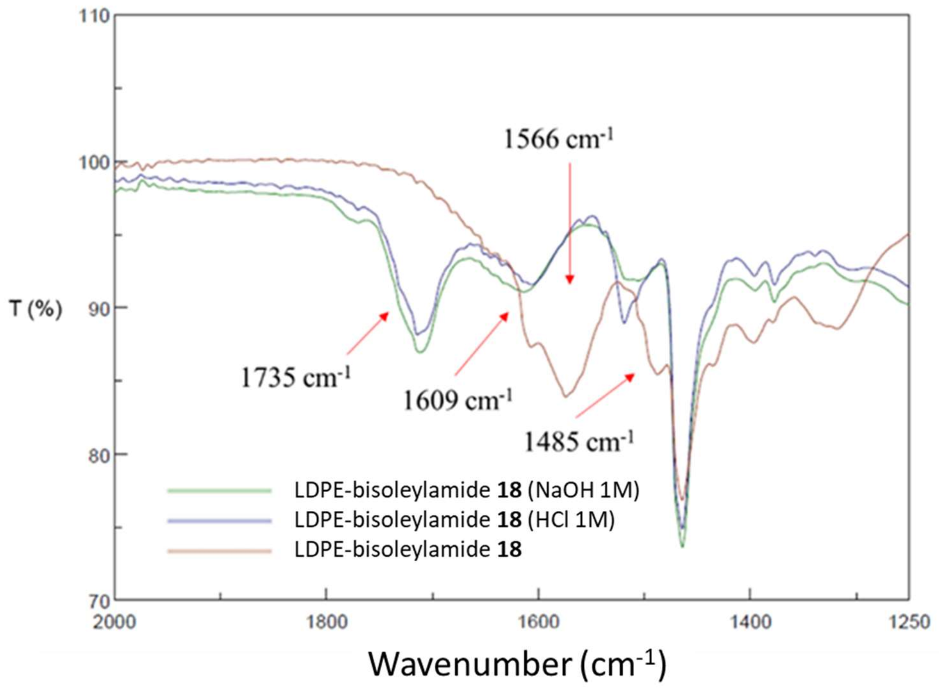

Figure S33 Fourier transformed infrared (FTIR) spectra of LDPE-bisoleylamide **18** after treatment with 1M NaOH or 1M HCl in boiling water (100 ºC) for 1 h.

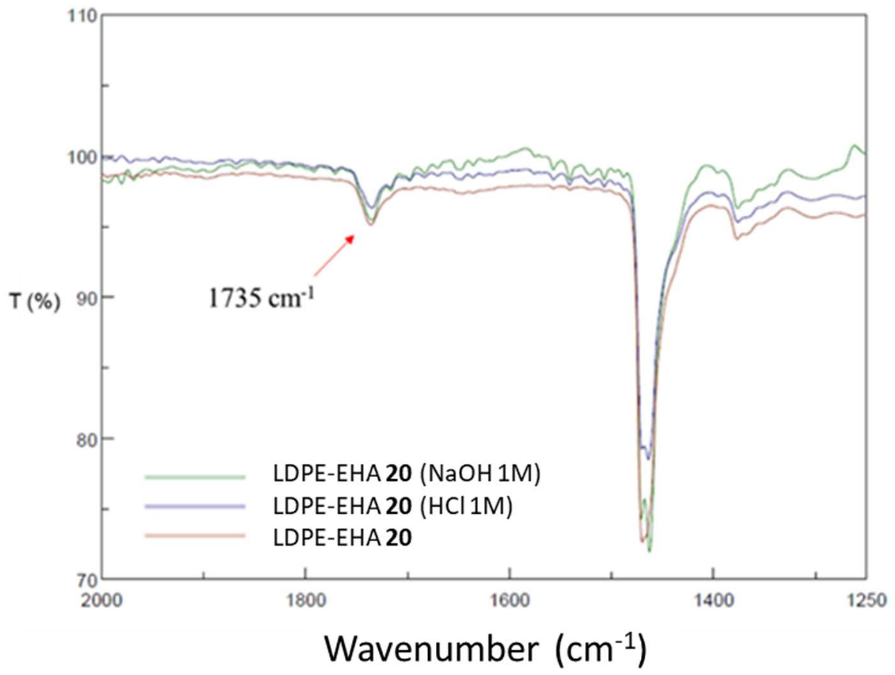

Figure S34 Fourier transformed infrared (FTIR) spectra of LDPE-EHA **20** after treatment with 1M NaOH or 1M HCl in boiling water (100 ºC) for 1 h.
